# Optimised LAMP allows single copy detection of 35Sp and NOSt in transgenic maize using Bioluminescent Assay in Real Time (BART)

**DOI:** 10.1038/s41598-018-36207-4

**Published:** 2018-12-04

**Authors:** Patrick Hardinge, Guy Kiddle, Laurence Tisi, James A. H. Murray

**Affiliations:** 1 Cardiff School of Biosciences, Biomedical Sciences Building, Museum Avenue, Cardiff, CF10 3AX UK; 2 ERBA MDX, Bartholomew Walk, Cambridgeshire Business Park, Ely, Cambridgeshire CB7 4EA UK

## Abstract

Loop-mediated amplification (LAMP) has been widely used to amplify and hence detect nucleic acid target sequences from various pathogens, viruses and genetic modifications. Two distinct types of primer are required for LAMP; hairpin-forming LAMP and displacement. High specificity arises from this use of multiple primers, but without optimal conditions for LAMP, sensitivity can be poor. We confirm here the importance of LAMP primer design, concentrations and ratios for efficient LAMP amplification. We further show that displacement primers are non-essential to the LAMP reaction at certain concentrations providing accelerating loop primers are present. We investigate various methods to quantify DNA extracts from GM maize certified reference materials to calculate the target copy numbers of template presented to the LAMP reaction, and show that LAMP can amplify transgenic promoter/terminator sequences in DNA extracted from various maize GM events using primers designed to target the 35S promoter (35Sp) or NOS terminator (NOSt) sequences, detection with both bioluminescence in real-time (BART) and fluorescent methods. With prior denaturation and HPLC grade LAMP primers single copy detection was achieved, showing that optimised LAMP conditions can be combined with BART for single copy targets, with simple and cost efficient light detection electronics over fluorescent alternatives.

## Introduction

Quantitative molecular diagnostics are required to assess the concentration of genetically modified material in food sources. Within the European Community, regulation 1830/2003 requires the appropriate labelling of food products where the percentage concentration of genetically modified material exceeds 0.9 percent. In California in 2012 the vote went against ‘Proposition 37’ which would have required mandatory labelling for all GMO contamination^1^. Low target number sensitivity is therefore needed, which in turn requires optimisation of the detection technique. Numerous techniques have been employed for GM detection^2^ with the majority centred on specific nucleic acid sequence determination. Novel techniques such as capillary electrophoresis with laser-induced fluorescence^3^ and biosensing with oligonucleotide conjugated gold nanoparticles^4^ can detect the cauliflower mosaic virus 35S promoter (CaMV 35Sp) at 0.1 percent GMO to wild type, but have not been widely adopted. Real time quantitative polymerase chain reaction (qPCR) has become the benchmark technique due to sensitivity and specificity, but there are challenges and costs to overcome for accurate GM quantitation^5^.

Isothermal amplification methods have also been used for GM detection^6,7^ with the potential benefit of lower cost instrumentation, since isothermal amplification of nucleic acid template operates at a single temperature without the requirement for thermocycling equipment. There are numerous described methods^8–10^ with redesigned or modified oligonucleotides, using either enzymatic strand displacement or RNA production to amplify the nucleic acid template. The various DNA based methods require strand invasion to generate accessible template before and during polymerisation to achieve exponential amplification.

**Figure 1:**
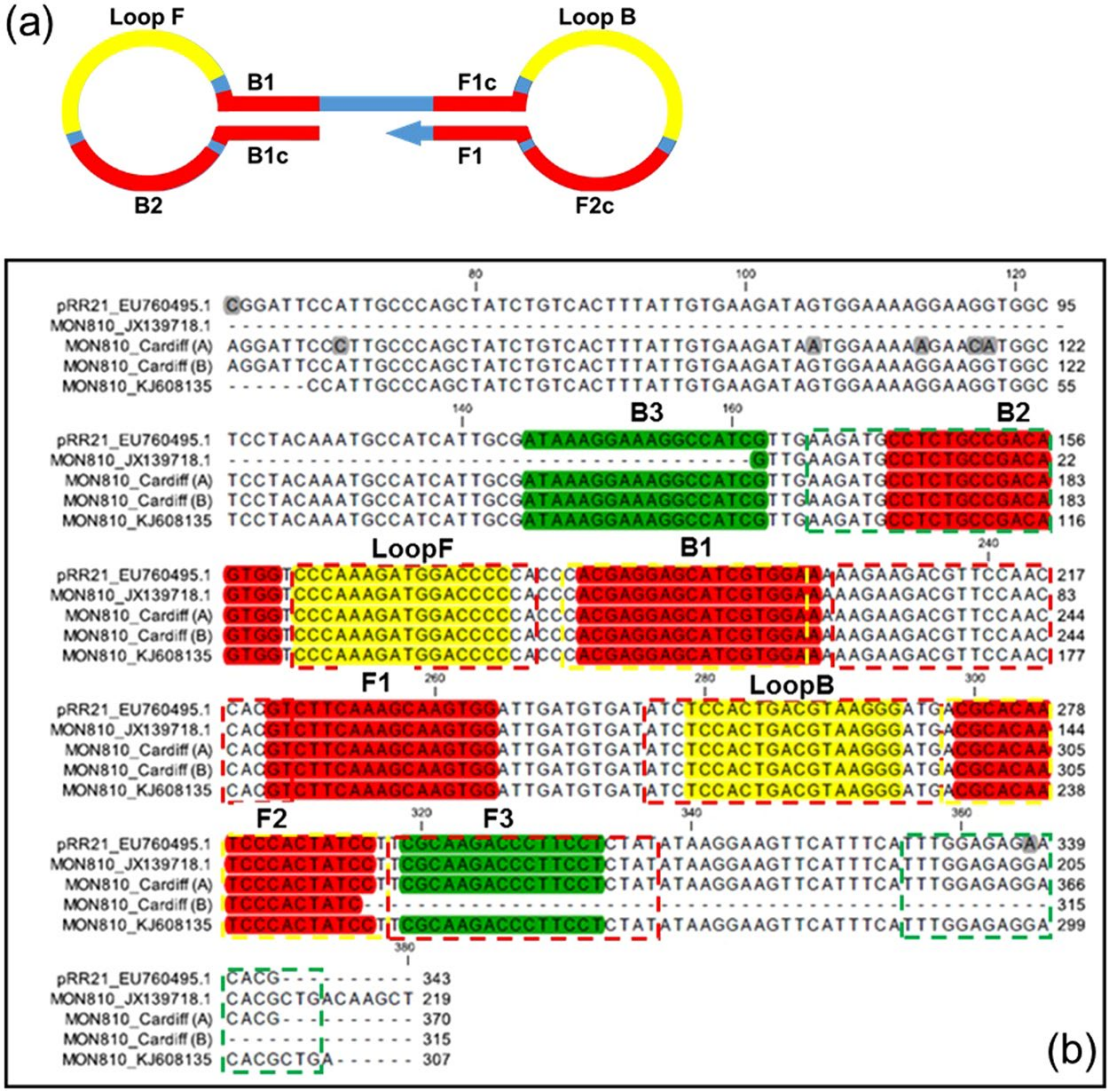
Sequencing data for MON810 35S promoter to show LAMP primer positions. (**a**) Positions of primer sequences on basic dumbbell amplicon produced after LAMP initiation. (**b**) New CaMV 35Sp sequence data from PCR amplified Mon810 transgene compared to GenBank EU760495.1, JX139718.1 and KJ608135 highlighting the partial nature of the 35S promoter sequence from JX139718.1. The alignments of the sequences are shown with the position of LAMP primers designed by Lee *et al.*^28^ and used by Kiddle *et al.* (“K-primers”), highlighted as follows; displacement primers denoted F3 and B3 are in green, loop primers denoted by F-Loop and B-Loop are in yellow and the hairpin-forming LAMP primers FIP and BIP composed of the two sequences F2, F1c and B2, B1c as indicated and highlighted in red. Primers used by Zahradnik *et al.* 2014 (“Z-primers”) are also indicated with dashed lines.

Loop-mediated isothermal amplification (LAMP) is a method that uses a strand displacing DNA polymerase and hairpin-forming oligonucleotide primers^11^. LAMP is simple, rapid and target specific, with greater robustness to inhibitory substances than PCR^6,12^. Two distinct types of primer are required for LAMP; hairpin-forming LAMP primers that are integral to the amplification, and displacement primers which are required during amplification initiation to generate the initial dumbbell structure that creates the basic amplicon. These primers are designed to target at least six template annealing positions, resulting in a high degree of specificity (Fig. 1a). Further primers can be used to accelerate the LAMP reaction; these are Loop primers binding within the dumbbell loops^13^ and STEM primers^14^ which anneal to the region between the hairpin loops.

The complex LAMP amplification mechanism is initiated by the hairpin-forming LAMP primers (forward and backward inner primers; FIP and BIP) by strand invasion of the double stranded DNA template. DNA polymerisation is initiated from these primers and the newly synthesised strands are displaced by primers designated F3 and B3. The absolute requirement for these displacement primers is generally assumed, but remains unclear and is addressed in the present work. The displaced ‘dumbbell’ structure with hairpin loops at each end is the structure required for cycling and elongation stages using the FIP and BIP primers. Typically LAMP amplification is conducted at 60 to 65 degrees with the length of assay time dependent on the reaction kinetics and concentration of the template. Amplification will proceed without the need for target denaturation, but there is evidence that pre-denaturation increases assay sensitivity^15^, supported further by results in this paper. By denaturing the double stranded DNA, the potentially limiting step of strand invasion by LAMP primers into the ‘breathing’ DNA is removed and the sensitivity thereby increased.

Nucleic acid amplification using LAMP can be detected in a number of ways. The first method exploited the production of inorganic pyrophosphate (PPi), a by-product of amplification, using the turbidity of magnesium pyrophosphate^16^ or in real-time^17^. Various fluorescence methods have been employed with calcein^18^ and nucleic acid intercalating dyes such as SYBR green, ethidium bromide and PicoGreen for end point detection of LAMP amplification after completion of reactions. Also colorimetric determinations have been shown^19^. Real-time detection of fluorescence has been described with SYBR green^20^, probe based methods such as DARQ^21^ and QUASR^22^ and here we demonstrate the effective use of SYTO9^23–25^. The bioluminescent assay in real time (BART)^26^ exploits the light emission produced by recombinant thermostable luciferase utilising the substrates luciferin and ATP. The ATP is produced from pyrophosphate in an ELIDA reaction (enzymatic luminometric inorganic pyrophosphate assay)^27^ whereby ATP-sulphurylase catalyses the reaction. The development of thermostable luciferase functional at 60 to 65 degrees makes BART possible [Gandelman *et al*.^26^].

BART detects nucleic acid amplification in a closed tube format, which reduces the risk of amplicon contamination. The light produced from each sample partition is recorded continuously and analysed by a simple CCD camera or photodiode detection system linked to a computer. A heat block is required at a single temperature. The BART profile for a positive sample is characterised by a peak of light at a particular time point at which the concentration of pyrophosphate inhibits the luciferase through the depletion of the substrate adenosine 5′phosphosulphate (APS). The time from the start of the assay to time-to-peak or Tmax is inversely proportional to the concentration of the original sample DNA template. LAMP amplification has been used successfully with BART due to the high concentrations of inorganic pyrophosphate produced enabling low nucleic acid copy number detection.

The sensitivity of LAMP based GM assays has been reported in several papers, including the detection of 10 copies of a marker in transgenic soya^28^, 10 copies of maize event markers^29^ and 10 copies for the detection of three GM rice events^30^. The possible sensitivity of LAMP to amplify single copies has seen some developments towards absolute quantification using digital LAMP^31–33^ using microfluidic devices.

However, poor sensitivity results for LAMP have also been reported recently^7^ with the detection of only 2 of 5 replicates for a 5 percent mass fraction of the transgene of maize event MON810 in wild type maize, corresponding to 770 copies per reaction based on the assumption of one transgene copy per genome equivalent. This work was in contrast to earlier work on the same target which reported a sensitivity of 50 copies (Kiddle *et al*.), and led to a discussion and response in the literature^34,35^. The poor sensitivity reported highlights the requirement for further analysis to establish the reasons underlying apparent differences in LAMP sensitivity at low target concentrations.

Here we analyse in detail the differences in the literature, and in resolving these clearly demonstrate how appropriately optimised LAMP reactions can readily amplify and hence detect the presence of a single transgene copy present in a reaction of DNA extracted from various maize GM events using primers designed against both 35S promoter (35Sp) and NOS terminator (NOSt) sequences. We show that the use of HPLC purified primers and the denaturation of genomic template is important in the reliable amplification of very low copy numbers. Furthermore we show that LAMP amplification can be detected with both bioluminescent and fluorescent real-time strategies with comparable results at single copy sensitivity.

## Results

### Optimisation of LAMP for high sensitivity

We investigated the reported absence of DNA sequence in the 35S promoter homologous to one of the Kiddle *et al*.^6^ and Lee *et al*.^28^ primers by aligning published sequences with our newly obtained sequence results for Mon810 and Bt11. Sequences for the 35S promoter in the plasmid vector pRR21 (GenBank EU760495.1) and in Mon810 (GenBank JX139718.1 and KJ608135) were compared to our sequence results for Mon810 (Fig. 1). The position of the LAMP primers used by Kiddle *et al*. are highlighted and labeled according to type, the primers designed by Zahradnik *et al*. are indicated by dashed lines. Sequence alignment for the displacement primer B3 shows that JX139718.1 is a partial sequence and the required sequence is present. The alignment for Bt11 (Fig. S1) also clearly demonstrates the presence of all sequences for primer binding.

**Figure 2:**
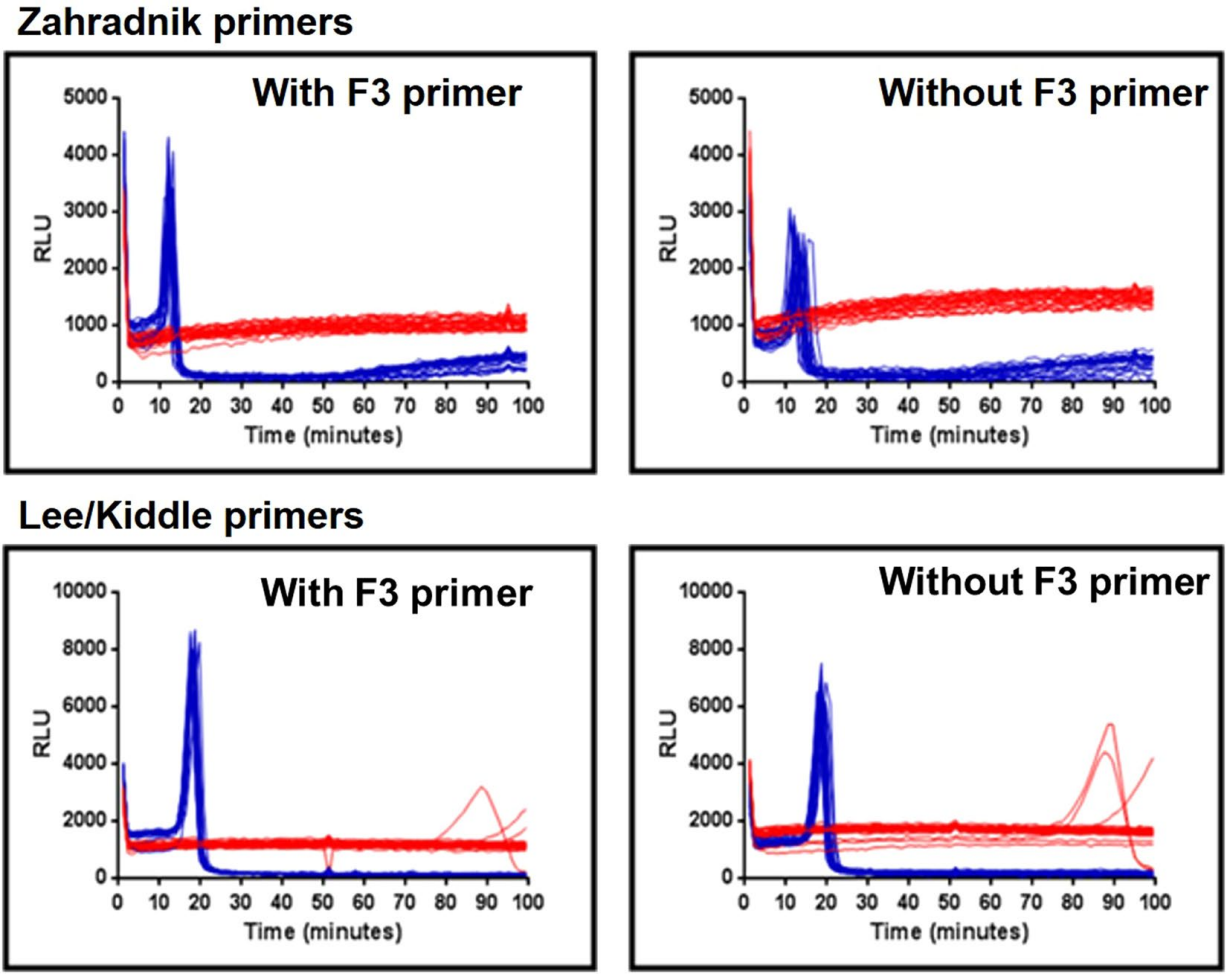
LAMP amplification without the F3 displacement primer. BART detection using (**a,b**) Z-primers and (**c,d**) K-primers to show LAMP amplification with or without the F3 displacement primer. Individual reaction partitions s were run in wells of microtitre plates (see Methods). Partitions containing DNA template (n = 24) are in blue with no template controls (NTCs) (n = 24) in red. Traces show the measured light output in relative light units (RLU) over time for each individual reaction partition. The peak of light in the positive samples is due to LAMP amplification (see Methods). Maize genomic DNA (prepared from commercial Mon810 seed) at a calculated 208 copies 35S promoter per partition and standard primers subject only to desalting were used.

The conclusion of Zahradnik *et al*.^7^ that the absence of a displacement primer sequence would necessarily have caused LAMP to fail was further investigated using both the primer sets used by Zahradnik (Z primers) and those used by Kiddle (K primers) to amplify 35S sequences of genomic DNA extracted from ground seeds from variety Mon810. The DNA quantity was calculated to contain 208 copies of the 35S target, assuming that the material was hemizygous and 1 ng of transgenic DNA is equivalent to 160 target copies (see Methods). BART reporting of amplification was used. Light peaks indicating amplification occurred within 10–20 minutes, with a delay of a few minutes when the F3 displacement primer was omitted (Fig. 2). The variation in amplification timing between replicates also increased when one displacement primer was omitted, and the peak of amplification signal was lower (Fig. 2d). Nevertheless, for all primer combinations, the amplification frequency was 100 percent whether or not primer F3 was present at a calculated presence of 208 copies of target per reaction. We conclude that LAMP amplification can proceed effectively on a genomic template in the absence of the F3 displacement primer using either 35S promoter LAMP primer set, but that the rate and reproducibility of the LAMP reaction were compromised by the removal of a displacement primer, suggesting this might affect sensitivity at extremely low copy number. Nevertheless, omission of one displacement primer does not prevent effective LAMP amplification on genomic samples at down to 200 copies per reaction.

**Figure 3:**
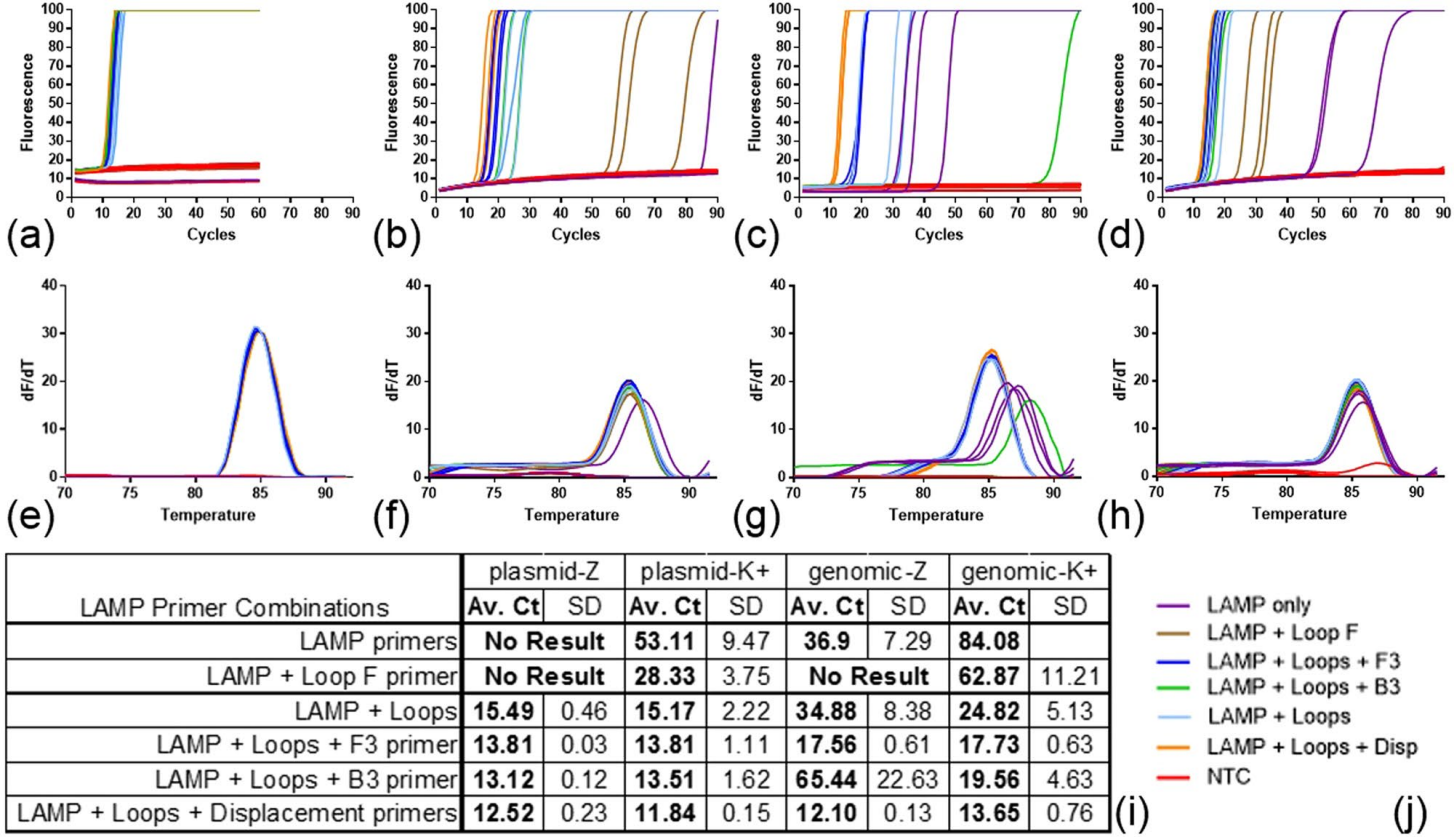
LAMP amplification without displacement primers. LAMP amplification with fluorescence detection with SYTO9 dye using Z primers and K+ primers for the 35S promoter of a linearised plasmid template and a native maize genomic template (prepared from commercial Mon810 seed); fluorescence and melt curve analysis for primer combinations (**j**) for: (**a,e**) Z primers with plasmid template, (**b,f**) Z primers with genomic template, (**c,g**) K+ primers with plasmid template, (**d,h**) K+ primers with genomic template. The Ct values were derived from threshold setting of 0.6 and the average and standard deviation from triplicate results displayed in table (**i**). The DNA concentration for the linearised plasmid template was 5000 copies per partition and 2078 copies per partition for the Mon810 template. Reactions were run on a RT-PCR machine (see Methods), and since LAMP is an isothermal technique “cycle” length (panels a–d) was equivalent to one minute; hence “Av. Ct” is time in minutes to detected amplification. Primer combinations; LAMP primers only in purple, LAMP primers with Loop F in brown, LAMP with both Loops and F3 in dark blue, LAMP with both Loops and B3 in green, LAMP with both Loops in light blue, all six primers in orange, NTCs for each primer combination in red. Amplicon melt curve analysis showed positive results with a consistent melt temperature (85 degrees) for the all six primers combination. SD: standard deviation.

To ascertain whether the reporter system used for LAMP amplification alters the observed sensitivity in marginal conditions when primers are omitted, whether both displacement primers can be omitted, and whether template type affects these results, the primer combination assay was conducted using SYTO 9 for fluorescent reporting of amplification. The results previously obtained with BART were replicated (Fig. 3) in that either displacement primer could be omitted using either genomic or linearised plasmid template without loss of LAMP amplification. Moreover, we found that LAMP amplification could proceed without any displacement primers albeit with reduced amplification efficiency, providing loop primers are present. For linearised plasmid this slowed down average Tmax from 12.52 to 15.49 and 11.84 to 15.17 minutes for Z-primers and K-primers respectively. Larger differences were observed with genomic template. Melt curve analysis showed LAMP amplicon formation with a melt temperature of 85 degrees for all positive results and no values for the no template control (NTC) and LAMP primer only reactions. These data confirm that displacement primers are not required for LAMP amplification at the copy numbers investigated, provided accelerating loop primers are present, and that results are independent of detection method.

**Figure 4:**
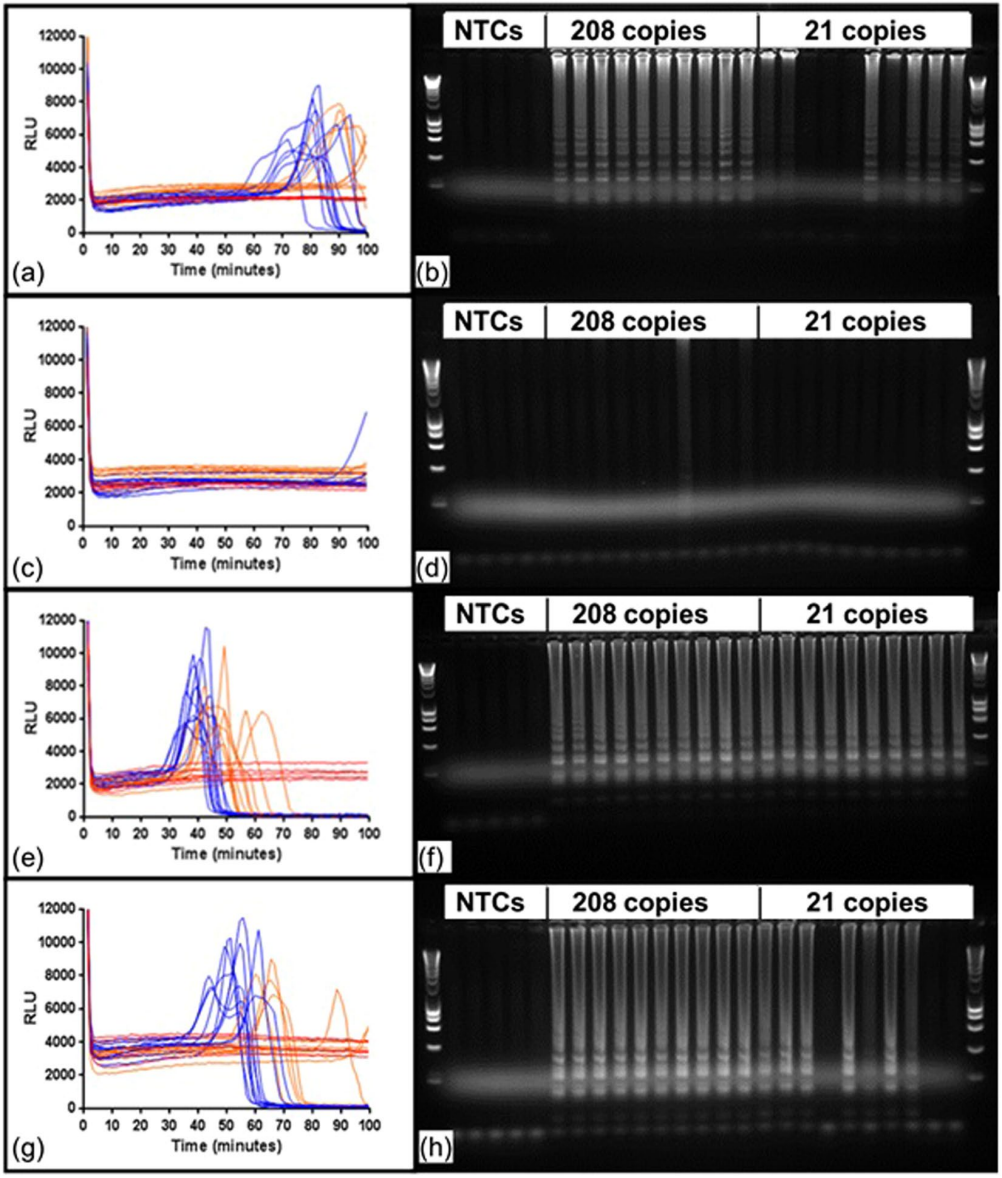
Two sets of 35Sp LAMP primers with Zahradnik and Kiddle primer concentrations and ratios. LAMP amplification using Z-primers and K-primers to the concentrations and primer ratios cited in Zahradnik *et al*.^35^ using BART real-time and agarose gel end-point detection of the 35S promoter in maize event Mon810. Note ladder pattern typical of LAMP amplifications. For the LAMP-BART charts of light output against time, 208 copies 35Sp per 5 microlitre are shown in blue, 21 copies are in orange and NTCs are shown in red. (**a**) Z primers with Zahradnik concentrations and ratio (**b**) agarose gel of LAMP-BART amplicons (**c**) K primers with Zahradnik concentrations and ratio (**d**) agarose gel of LAMP-BART amplicons (**e**) Z-primers with Kiddle concentrations and ratio (**f**) agarose gel of LAMP-BART amplicons (**g**) K primers with Kiddle concentrations and ratio (**h**) agarose gel of LAMP-BART amplicons. Figs S7 and S8 show the original full length agarose gels.

Having investigated the primer requirements for 35S promoter detection, we analysed the effect of LAMP primer ratios on assay kinetics and sensitivity. Zahradnik *et al*.^7^ used the ratio of primers LAMP: Loop: displacement of 4:1:2 at concentrations of 0.8, 0.2 and 0.4 micromolar respectively. In contrast, Kiddle *et al*. used the ratio of 4:2:1 of LAMP: Loop: displacement at concentrations of 1.6, 0.8 and 0.4 micromolar. Since we did not observe amplification differences when halving these concentrations, we reduced the concentration of the K primers to 0.8:0.4:0.2 to allow direct comparison with Zahradnik *et al*. at the same LAMP primer concentration. Using the Zahradnik ratios with the Z LAMP 35S primer sets at two concentrations of native DNA from maize event Mon810, the assay was slow for both concentrations of template (Fig. 4). LAMP-BART reactions were confirmed by endpoint agarose gel analysis showing amplification was successful for all reactions containing 208 copies per reaction, and for 60 percent of reactions containing 21 copies (Fig. 4). We conclude that LAMP-BART has comparable sensitivity to the end-point method. However the K-primers showed no amplification of template at either concentration using the Zahradnik ratios. This failure replicates the observation of Zahradnik *et al*.^7^ that they could not achieve LAMP amplification with K LAMP primers for the detection of maize event Mon810 (prepared from commercial seed).

However, altering the ratio of LAMP primers to 4:2:1 of LAMP: Loop: displacement as used by Kiddle *et al*. at the same concentrations of 8, 4 and 2 micromolar dramatically improved all results. Faster reaction times were seen with the Z primers (Fig. 4e,) than previously observed (Fig. 4) and amplification with K primers was also seen, indicating that this primer ratio is more favourable. The sensitivity of the LAMP-BART and the LAMP-endpoint assays was improved with a 100 percent amplification frequency for both 208 and 21 copies with the Z-primers and 100 percent at the higher concentration with K-primers and c. 65 percent at the lower concentration.

However, these results explain the data previously reported (Zahradnik *et al*.^7^), and demonstrate the impact of and requirement for optimised LAMP primer concentrations and ratios. These results also indicate that the Z-primer set appears to have superior performance giving faster and more reliable amplification at lower target concentrations, but the optimised primer ratio of Kiddle *et al*. works better with both primer sets.

### Single Copy Detection

**Figure 5:**
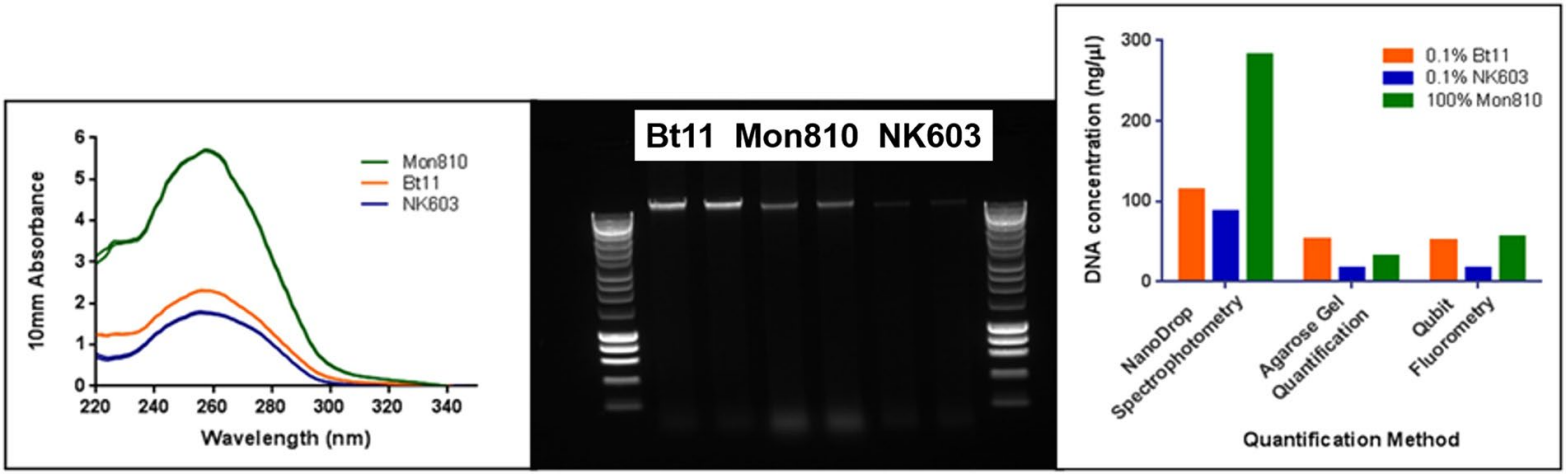
Quantification of maize genomic DNA. Three methods for determining the DNA concentration of the genomic DNA extracts from maize events Bt11, Mon810 and NK603 were compared. DNA extracts were derived from a similar quantity of starting material (40 milligrams as defined by the extraction protocol with multiple extracts combined to exceed the 100 milligrams ERM minimum recommendation) and extracted to the same protocol. Left: NanoDrop spectrophotometer results for each maize event in duplicate (the 260:280 nanometre ratios for all samples ranged from 1.71 to 1.81 and the ratio 260:230 nanometre from 1.69 to 2.59, Bt11 concentration 114.3 nanograms per microlitre); Centre: duplicate loading of DNA from each maize event on an agarose gel (full size gel image shown in Fig. S9) compared to ladder of defined DNA concentrations showing intact genomics DNA of large fragment size (Bt11 concentration 52.0 nanograms per microlitre); Right the three quantification methods compared (Qubit determined Bt11 concentration 51.4 nanograms per microlitre).

We next sought to make use of optimised assays to determine whether single target detection could reliably be achieved. The calculation of the dilution required to achieve an average of one target DNA copy per reaction partition necessitates the accurate determination of DNA concentration from genomic DNA samples prepared from standard Certified Reference Material (CRM) maize meal. This must then be converted to DNA target copies using a calculation based on the zygosity of the CRM which is normally of hybrid origin, contribution of different tissues to the seed and any duplication of sequences within the transgenes present^36,37^. Our previous results suggested that spectrophotometric measurement (Nanodrop) can over-estimate the concentration of maize genomic DNA purified using standard kits when compared to agarose gel quantification (Kiddle *et al*.^34^). To extend this analysis, we compared Qubit fluorimetry with NanoDrop spectrophotometry and agarose gel band intensity methods for quantifying DNA purified from maize events Mon810, Bt11 and NK603 (Fig. 5), and found wide variation between spectrophotometric and other methods dependent on sample.

**Figure 6:**
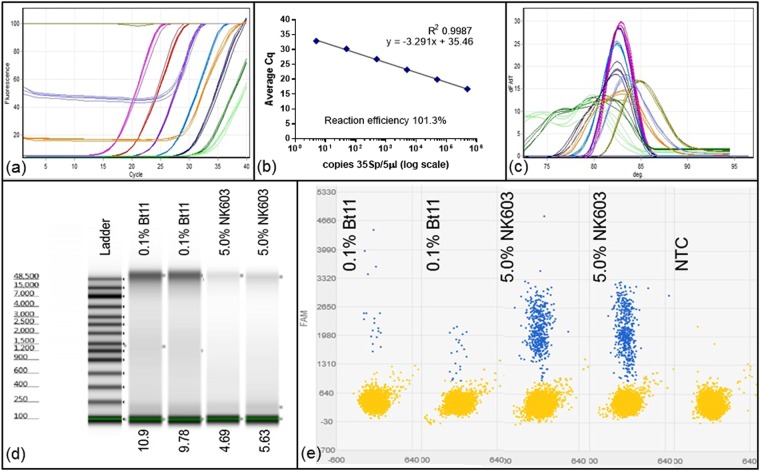
Copy number determination using quantitative PCR against standard curve, Quant Studio 3D digital PCR and TapeStation analysis. (**a**) Quantitation of 0.1 percent Certified Reference Material (CRM) Bt11 DNA extract and known dilutions of linearised plasmid pART7[49] containing the 35Sp sequence incrementally from 500000 copies (pink) to 5 copies per 5 microlitres (dark green). Dilutions of the 0.1 percent Bt11 extract were; mid-green for undiluted, mid-blue for 1:2 dilution and light brown for 1:5 dilution. NTCs: pale green. (**b**) Derived standard curve using pART7 data. Standard curve of this linear plasmid DNA has calculated PCR efficiency of 101.3 percent with R2 = 0.9987. (**c**) Melt curve analysis of amplicons from (**a**). (**d**) TapeStation analysis of genomic DNA for undiluted 0.1 percent Bt11 and 5.0 percent NK603 CRM DNA extracts using an Agilent genomic DNA ScreenTape. (**e**) Quant Studio 3D digital PCR results using 35Sp primers with a TaqMan probe (see Methods) showing negative partitions in yellow and positive partitions in blue from which the concentration of the samples are calculated (Table 1).

We further investigated quantification using a DNA extract from maize event Bt11 to compare DNA measurement by fluorimetry with Agilent TapeStation using a genomic tape, and direct copy number determinations by qPCR, digital PCR (dPCR) using a TaqMan probe in a Quant Studio 3D assay. Quantitative PCR quantification was calibrated against a defined dilution of linearised plasmid carrying the 35S promoter in the range 5 to 500000 copies per 5 microlitres (Fig. 6). The dilutions of the Bt11 genomic sample were quantified against these curves. For the native genomic DNA, the background fluorescence of the undiluted and the 1:2 diluted samples were high; the calculated DNA concentration of the undiluted Bt11 sample taken from a 1: 5 dilution was 12.2 nanograms per microlitre. Replicate qPCR results are shown in Fig. S3.

Results are presented in Fig. 6 for samples of DNA extracted from 0.1 percent Bt11 sample and 5.0 percent NK603 CRM, and summarised in Table 1 compared to data derived from known dilutions of plasmid pART7 carrying the same target sequence. We conclude that the NanoDrop overestimated the concentration of our sample when compared to other quantification techniques. There was however excellent agreement between DNA measurements by the Qubit fluorimeter and Agilent Tapestation. Copy numbers of 35S sequences for the Bt11 sample were converted to calculate DNA equivalents and values of 7.04 nanograms per microlitre for qPCR and 18.62 nanograms per microlitre for digital PCR were obtained. We note that the upper figure of 18.62 nanograms per microlitre for digital PCR may be subject to reduced reliability due to the low number of positive partitions on the QuantStudio 3D digital PCR chip, although the overestimate of DNA concentration was also apparent with the 5 percent NK603 extract. Both the qPCR and dPCR methods quantify based on copies of the 35S promoter sequence. The conversion of these values to nanograms of DNA are corrected for hemizygosity of the GM material, the contribution of tissues of different ploidy and parental origin to the sample, and the two copies of the 35S target sequence present in Bt11. The Qubit fluorimeter measurement was a convenient method to calculate single target copy number for our LAMP sensitivity investigations, based on the closest value to the average of the four techniques (Table 1).

**Table 1:** Summary of quantification results.

Quantification Method (native 5.0 percent NK603 extract)	DNA conc (ng/microlitre)
NanoDrop spectrophotometer	**30.87** +/− 2.23
Qubit fluorimeter dsDNA BR kit	**4.74** +/− 0.08
Agilent Tapestation genomic tape	**5.16** +/− 0.66
QuantStudio 3D digital PCR 35Sp TaqMan assay	**9.88** +/− 0.11
**Quantification Method (native 0.1 percent Bt11 extract)**	**DNA conc (ng/microlitre)**
Qubit fluorimeter dsDNA BR kit	**12.30** +/− 0.07
Agilent Tapestation genomic tape	**10.34** +/− 0.79
QuantStudio 3D digital PCR 35Sp TaqMan assay	**18.62** +/− 1.96
Quantitative PCR 35Sp assay	**7.04** +/− 1.63

Summary of quantification of the 5.0 percent NK603 and 0.1 percent Bt11 native maize DNA extracts by various quantification techniques.

Qubit fluorimeter quantifications were used to calculate the dilutions of 0.1 percent Bt11 maize DNA extract to give the equivalent of single copy or less of target sequence per reaction. Here we also took into account the effect on target copy number of denaturing the template before addition to the reactants, since an originally double stranded template yields two amplifiable molecules on denaturation, and these are then assumed to distribute independently. We also assumed that any duplicated sequences, such as the two 35S promoter sequences in Bt11, distribute independently.

**Figure 7:**
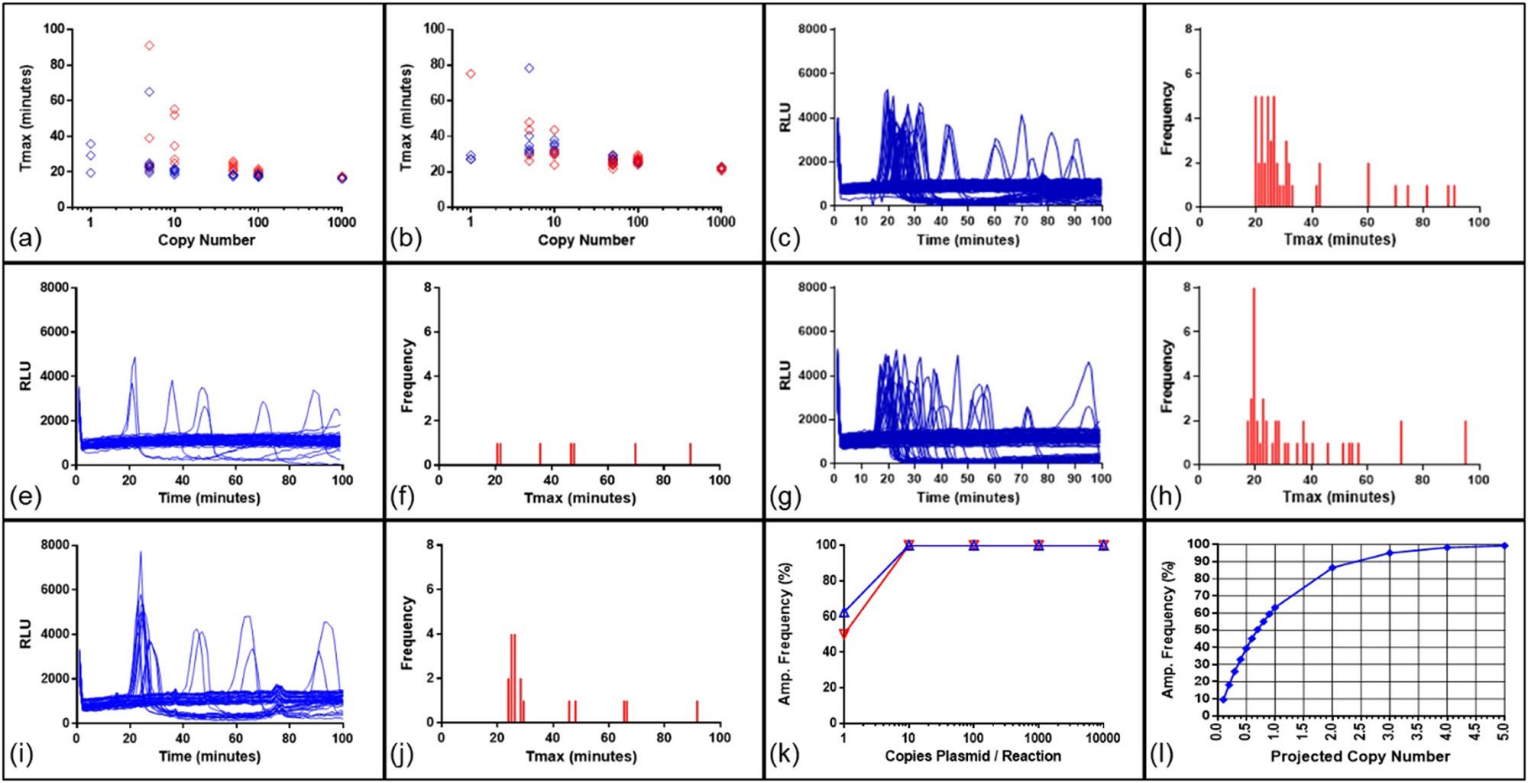
Optimisation of the 35Sp K primer set. LAMP-BART time-to-peak (Tmax) results shown plotted against copy number of a linearised plasmid for; (**a**) comparison between K-primers in red and K+ primers in blue; (**b**) comparison between desalted primers in red and HPLC purified primers in blue. 8 replicates were run in each case. (**c,g**) Comparison between 0.5x concentration of K+ primers (0.8 micromolar LAMP, 0.4 micromolar Loop and 0.2 micromolar displacement primers) for 1 copy per partition using 96 replicates and LAMP-BART detection of linearised plasmid template with standard concentration of K+ primers described in Kiddle *et al*. (1.6 micromolar LAMP, 0.8 micromolar Loop and 0.4 micromolar displacement primers) (d,h) shows frequency histograms for Tmax distribution in (**c,g**). Comparison between native and denatured template with K+ primers: (**e,f**) LAMP-BART of 4 copies per partition native maize genomic DNA (Mon810), (**i,j**) LAMP-BART of 4 copies per partition denatured genomic template. 48 replicates were run in each case. (**k**) Amplification frequency for linear plasmid template using 1–104 copies; native in blue and denatured in red. (**l)** Predicted amplification frequency against copy number derived from Ucount^TM^ software for 0–5 copies.

To maximise amplification, and since we had observed that amplification of the K primers was less effective than Z primers, we sought to optimise further the K primer set. Changes were made to the length, GC content and melt temperature of the displacement primers and the FIP primer. Comparison of the optimised K+ primers showed improved performance to the K primers in sensitivity, reproducibility and reaction kinetics (Fig. 7a). We further observed that primer quality affects amplification performance and the use of HPLC purified primers gave further performance enhancement with reduced replicate variation (Fig. 7b) when compared to desalted primers. The concentration of K+-primers in LAMP-BART assays was compared using the concentration described by Kiddle *et al*. (Fig. 7g,h) and halved concentrations (Fig. 7c,d). The amplification frequency for these two 96 partition single copy assays was 44 percent and 47 percent with average Tmax values of 33.65 and 33.57 minutes respectively. We concluded that the reduced primer concentration does not impact on LAMP-BART sensitivity and assay kinetics.

To understand the effect of template denaturation on linear plasmid and genomic DNA templates, samples calculated to contain 4 double stranded copies per partition of maize Mon810 genomic DNA were amplified with K+ primers (Fig. 7e,f for native and i,j for denatured). This resulted in an increase in positive partitions of 14.6 percent to 37.5 percent in response to DNA denaturation. We note that part of this improvement may be due to increasing the number of independently distributing molecules, although the improvement to amplification frequency was not observed with linearised plasmid template (Fig. 7k), as 100 percent amplification was seen at 10 copies per partition and above, and 62.5 percent and 50 percent for native and denatured respectively at single copy level; the difference between which is likely not significant. The genomic DNA denaturation may increase the amount of cryptic priming sites and therefore the amount of non-specific displacement priming. Moreover the denatured linear plasmid DNA is more likely to re-anneal than genomic DNA.

Having established optimised conditions, the sensitivity of LAMP-BART for near-single copy detection of DNA from maize event Bt11 was assessed using 35S promoter and NOS terminator primers for the same GM event.

**Figure 8:**
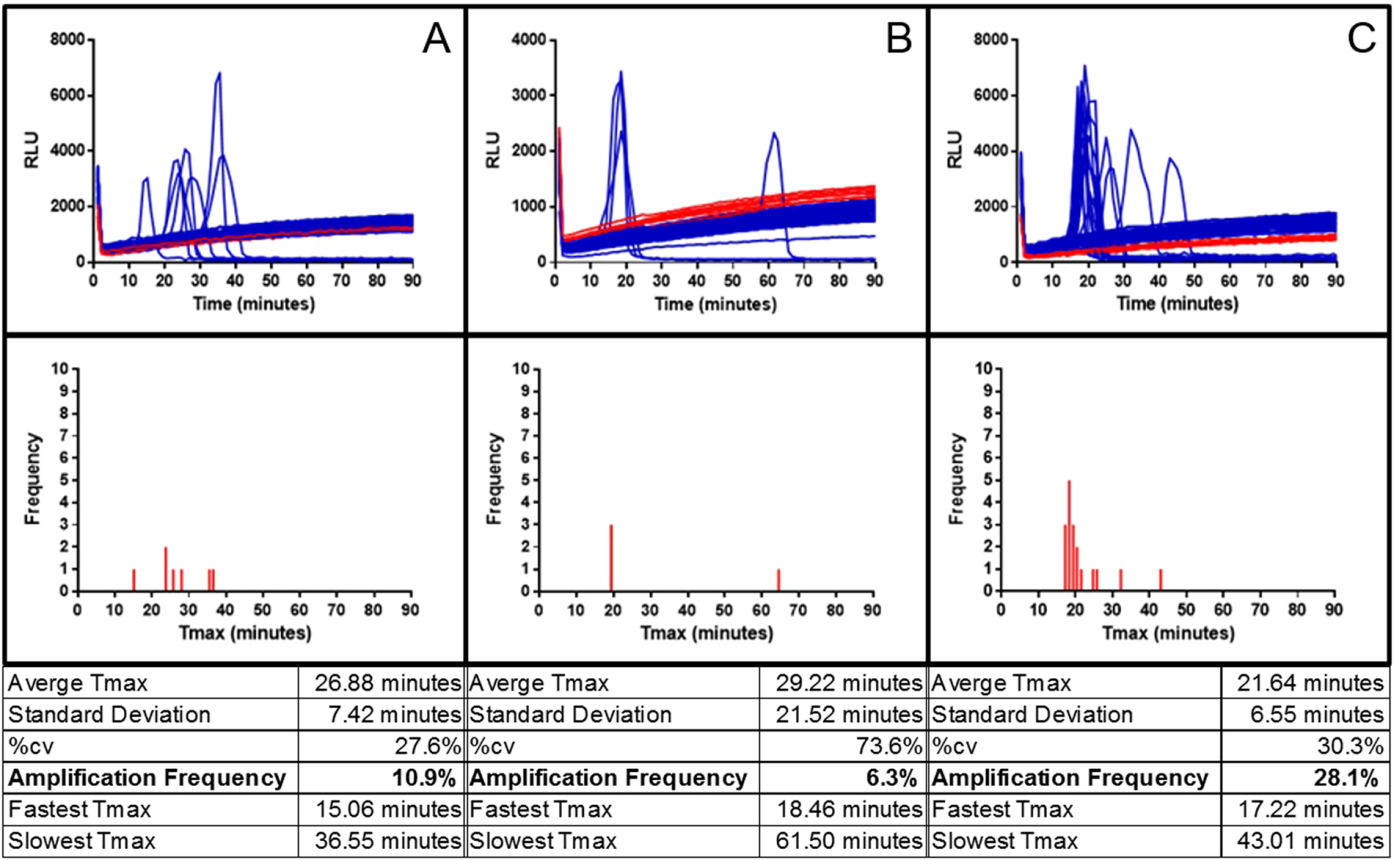
LAMP-BART detection of 0.8 copies of 35S promoter or NOS terminator sequences in denatured maize genomic 0.1 percent Bt11 DNA extract. Column (**A**) K+ primer assay; column (**B**) Z primers; column (**C**) NOSt primer assay. The top row shows traces from replicate partitions of LAMP-BART assays for 0.8 copies per partition (n = 64) in blue and the NTCs (n = 8) in red. The frequency distribution of LAMP-BART Tmax time-to-peak results for each assay is below with a summary analysis table. HPLC grade primers and fast Bst v2.0 warm start polymerase were used for all three sets.

Three replicate sets of assays were carried out with 64 test partitions each with a calculated mean 0.8 copies of denatured target sequence of 0.1 percent Bt11, plus 8 NTC partitions. Targeting the 35S promoter with K+ primers provided an overall mean value of 13.3 percent of partitions showing amplification (SD 3.3percent; Supplementary Fig. S4; representative example showing 7 positive partitions from 64 [10.9 percent] in Fig. 8). Using Z primers also targeted to the 35S promoter a mean of 4.7 percent, (2.3 percent SD; example in Fig. 8 shows 6.3 percent positive samples). Targeting the NOSt sequence, which is part of the same GM event in Bt11, showed a frequency of mean 30.5 percent, (3.3 percent SD; example shown Fig. 8 with 28.1 percent). We noted that the NOSt targeting assay produced a faster average Tmax and lower variation between replicates than the two 35Sp assays. We conclude that LAMP-BART assays can detect single copies of denatured genomic DNA target.

The same maize DNA extract dilution reaction was amplified, but with the detection provided by SYTO9 dye on a qPCR platform.

**Figure 9:**
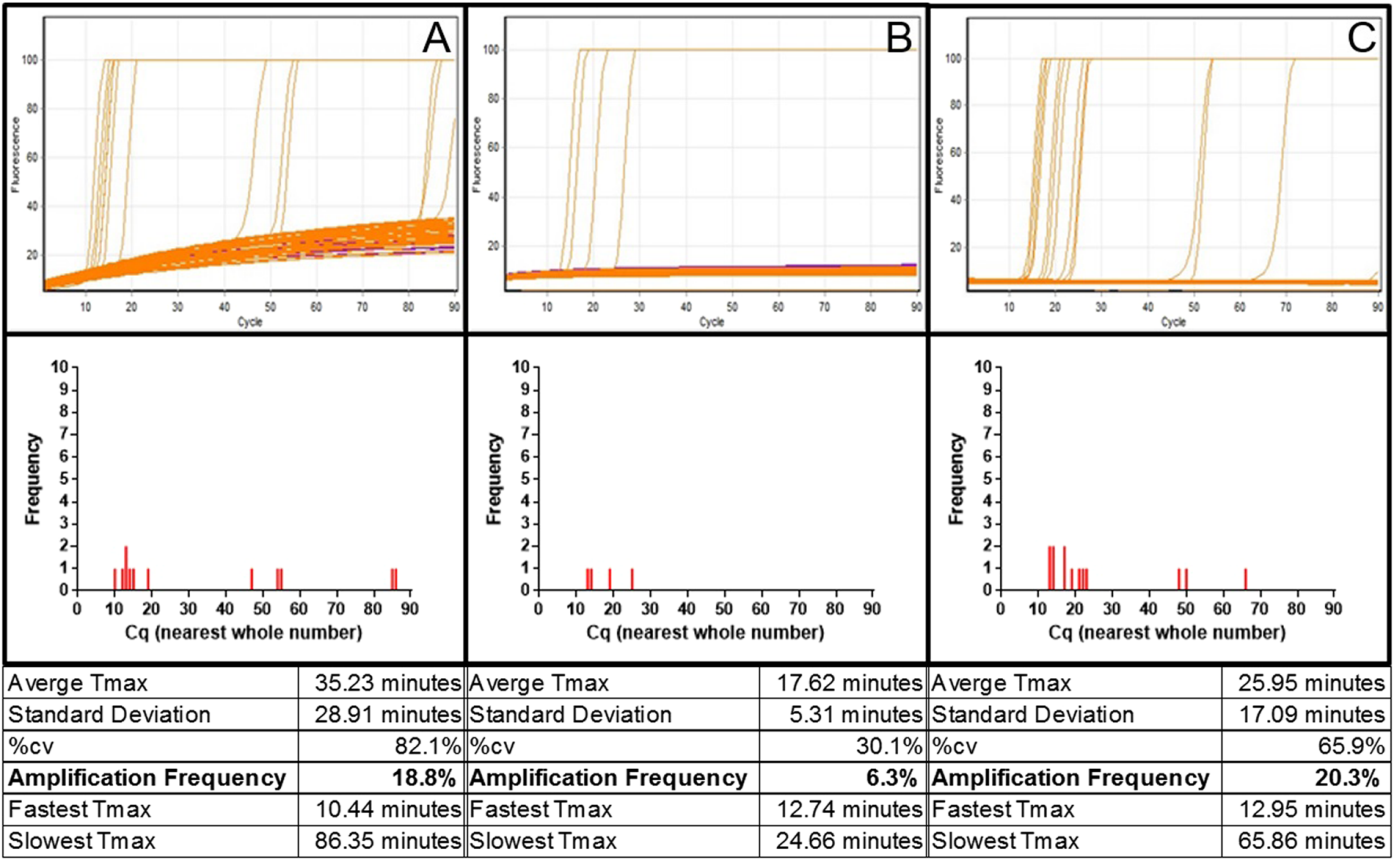
Fluorescent LAMP detection of 0.8 copies of 35S promoter or NOS terminator sequences in denatured maize genomic 0.1 percent Bt11 DNA extract. Column (**A**) K+ 35S primer assay; column (**B**) Z 35S primers; column (**C**) NOSt primer assay. Top row: fluorescent LAMP assays with calculated average 0.8 target DNA molecules per partition (n = 64) in orange and the NTCs (n = 8) in red. The frequency distribution of fluorescent LAMP amplification timing for each assay is below, with a summary table (each nominal cycle was 1 minute).

Replicate fluorescent LAMP assays targeting the 35S promoter with Z primers showed a frequency mean of 11.0 percent, 6.6 percent SD (Fig. 9; example assay set shown with 6.3 percent, Supplementary Fig. S5 shows 15.6 percent), broadly similar to the equivalent LAMP BART assay shown in Fig. 8(b). Timing of amplification was variable as previously observed using LAMP-BART on these low copy number assays, indicating this is a feature of LAMP, rather than the detection method used. Similar results were observed with the other two assays; the amplification frequency for the K+ primers for this assay had a mean of 12.5 percent, 5.7 percent SD (Fig. 9b, 18.8 percent in this set), and using NOSt primers had a mean of 28.6 percent; 7.2 percent SD (Fig. 9c; 20.3 percent, Supplementary Fig. S6; 32.8 percent and 32.8 percent), again producing a higher amplification frequency from the 0.1 percent Bt11 maize target than the 35S primers.

We can conclude that single copy detection can be achieved with LAMP using optimal conditions and that BART and fluorescent reporting of the amplification of single copies of denatured genomic DNA are similar in sensitivity.

## Discussion

Whilst controversy exists on the sensitivity of LAMP to amplify low copy number targets in the literature (Kiddle *et al*. and Zahradnik *et al*.)^6,7^, here we demonstrate clearly that LAMP amplification of genomic DNA targets is effective, and show that the design, ratios and concentrations of LAMP primers are key factors in determining LAMP sensitivity. Specifically Zahradnik *et al*.^35^ state that the sensitivity of LAMP targeting the cauliflower mosaic virus (CaMV) 35S promoter [Kiddle *et al*.; Zahradnik *et al*.]^34,35^ previously reported by Kiddle *et al*.^6^ was unattainable. To seek to resolve these discrepancies, we analysed all aspects of the reaction.

Firstly we used the Promega Wizard genomic DNA purification kit for our DNA extractions. In contrast Zahradnik *et al*. extracted maize DNA using an optimized cetyltrimethylammonium bromide (CTAB) protocol. Kiddle *et al*. showed improved sensitivity and reproducibility with the Promega Wizard kit compared to a CTAB protocol for maize extraction for LAMP amplification and detection with BART^6^.

The two papers make use of different primer sets (Figs S2 and 1b) and different primer ratios. However, in Zahradnik *et al*., a ‘theoretical and experimental’ problem was attributed to the 35S promoter LAMP assay of Kiddle *et al*.^6^ and Lee *et al*.^28^ due to the reported absence of sequence in the 35S promoter homologous to one of Kiddle’s primers, based on sequence from Spalinskas *et al*.^38^ (GenBank accession number JX139718.1). If correct, this would remove the binding site for the B3 displacement primer. Other published sequences for the 35S promoter in the plasmid vector pRR21 (GenBank EU760495.1) and two published sequences for Mon810 (GenBank JX139718.1 and KJ608135) were therefore compared to our newly obtained sequence results for Mon810, clearly demonstrating the presence of all primer sequences in both the standard 35S promoter and Mon810 T-DNA (Fig. 1). We concluded that absence of cognate sequence does not explain the differences between primer sets reported by Zahradnik *et al*.^7^. Additional newly obtained sequence results for Bt11 are shown in Fig. S1.

The combination of primers required for LAMP, including the LAMP primers themselves, displacement and loop primers makes for complex primer design, and two designed primer sets for the CaMV 35S promoter sequence may differ in efficiency and propensity for non-specific primer interaction. Accurate sequence data is of course required to confirm alignment with primers as a partial sequence can lead to inaccurate conclusions and attention should be brought to the presence of single nucleotide polymorphisms in the 35S promoter sequence^39,40^, when considering LAMP primer design. False negative results can result from poorly chosen primers and sub-optimal assay conditions leading to negative reports about the efficacy of LAMP for nucleic acid detection at low copy number. This was shown with the two sets of 35Sp LAMP primers with suboptimal primer ratios. We show that primer ratios, primer concentrations and reagent composition and concentrations markedly improved the amplification frequency with endpoint detection of the LAMP assays when using the reaction conditions specified in Kiddle *et al*.^6^. The concentrations of FIP and BIP primers were identical for the two ratios, however twice the concentration of displacement primers and half the concentration of Loop primers was used in the Zahradnik *et al*.^7^ ratio. There may be a dual impact of this on amplification kinetics; a sub-optimal concentration of Loop primers may reduce the rate of priming from Loops with concomitant reduction of pyrophosphate/DNA production, and the excess of displacement primers may occlude the potential of LAMP primers to invade and initiate amplification.

We further showed that LAMP amplification can proceed without either or both displacement primers, although the resulting amplification is sub-optimal leading to slower reaction times. It is unclear whether assay sensitivity at very low copy numbers would be compromised and this would require further investigation. Since LAMP amplification can proceed without displacement primers, this might suggest that the initiation of amplification can use an alternative mechanism to displace the hairpin-forming LAMP primers perhaps linked to the energetics of loop formation. This observation could be useful in the design and optimisation of LAMP primers in screening for non-specific primer interactions from the hairpin-forming primers independently of displacement primers.

A number of routinely used quantification techniques were compared to determine DNA concentration and target copy number either directly or by calculation. Measurement derived from the NanoDrop spectrophotometer were higher than the other methods for these samples, presumably due to residual RNA contaminants and other UV absorbing compounds interfering with measurement accuracy. The other quantification techniques provided results within a two fold range, and in particular Very similar results were obtained for fluorescence and Agilent TapeStation determinations of DNA concentration. The highest value in the range was derived from copy number determination by digital PCR with 35S promoter TaqMan primers. As this result is from absolute quantification this might be considered to represent a benchmark value (assuming 100 percent amplification efficiency). However the accuracy of this technique for quantification may have been compromised by the low number of positive results (36 and 41) from over 17000 used partitions. We took the value from the fluorimeter as the nearest to the average of the four techniques (digital PCR, Agilent TapeStation, fluorimetry and qPCR) which was the value from the fluorimeter. A similar approach was used by Holden *et al*. (2010) with PicoGreen binding.

Denaturing genomic DNA before LAMP amplification enhanced the detection of low copy numbers of the transgene 35S promoter. Without denaturation, LAMP amplification is initiated by primers invading the double stranded DNA and relies on the DNA ‘breathing’ to allow primers access. Denatured DNA has the benefit of being single stranded allowing primers to bind more readily. The increase in amplification frequency suggests that the initiation of LAMP amplification becomes less of a limiting step. The size and complexity of genomic DNA may be factors in the improvement from denaturation seen with genomic DNA when compared to linear plasmid DNA. However, we also note that prior denaturation presumably doubles the number of independently distributing molecules since both DNA strands are capable of initiating amplification, and our calculations take this into consideration. We propose that the normal practice of considering the number of double stranded DNA molecules needs to be revised when considering pre-denatured samples then analysed by digital or single copy techniques.

In the calculation of copy number for the CRM Bt11 and NK603 and for DNA prepared from our own seed stock of Mon810, we used the simulations of GM content from Trifa and Zhang^36^. Their calculations showed that the hybrid seed used for CRM material will carry a hemizygous GM locus derived from the transgenic male parent (as in the case of both these maize events). However, due to the seed being composed of diploid embryo and triploid endosperm (comprising two maternal and one paternal genome) will have a GM content of 160.4 copies per nanogram using the commonly accepted haploid maize genome size of 2.6 picograms. Homozygous transgenic maize contains 385 copies per nanogram of DNA. Our calculations of copy number reflect this reduction. There is further uncertainty with the CRM materials, which are composed of mixtures of ground seeds from GM and non-GM sources, since environmental factors during growth such as moisture content and temperature can affect DNA contents of the different ground material^41,42^. The certified value for the CRM based on grams per kilogram of GMO material of our sample European Reference Material supplied by the Joint Research Centre was reported to be 9.8 grams per kilograms with an Uncertainty of 0.9 grams per kilogram. As an example, an uncertainty of 2.1 grams per kilogram was assigned to a 5 percent Bt11 CRM with a certified value of 48.9 grams per kilogram at a 95 percent confidence level. Trapmann^43^ presents analysis to show that when calculating DNA content based on copy number determination, the relative expanded uncertainty associated with the measurement increased two-fold.

Both BART and fluorescence methods provided similar amplification frequencies in single copy detection with all three LAMP primer sets and both target sequences of the Bt11 sample. The highest amplification frequencies were for the K+ 35S primers and NOS terminator assays with both detection methods. Importantly for the LAMP detection strategies, similar amplification frequencies were observed with both LAMP-BART and fluorescent LAMP, which tracked the amplification in real time enabling a shorter assay time in comparison to visual end-point detection, also without the risk of contamination from opening tubes post-amplification.

In our assays with a calculated mean copy number of 0.8 per reaction, and assuming that the every DNA target molecule can initiate and is amplified with 100 percent efficiency, the calculated amplification frequency of multiple replicate assays should be 55 percent^44,45^. This frequency was not achieved using any primer set, and actual results varied between 6–30 percent for different primer sets; equivalent to an average copy number per partition of 0.1–0.5 (based on projections in Fig. 7(l)). There is variation in amplification frequency between the two assays targeting the 35S promoter as well as with the assay for the NOS terminator sequence in the same template, which indicates the importance of LAMP primer design to optimise the frequency of amplification. However, our assumption of the independent distribution of the two 35S promoter sequences present in the Bt11 transgene which are in fact contained on a single DNA fragment of 3–4 kb is likely to be true only in a subset of cases where the DNA is fragmented between these sites. Since our genomic DNA size is significantly larger than this (Fig. 6d), this is probably not correct, which would lead to an overestimate of effective copy number by a factor of up to 2. Together with measurement uncertainties explored in the previous paragraph, it is plausible that the best primer sets (K+ for 35S and the NOS terminator set) are achieving close to 100 percent target amplification.

Here we have demonstrated efficient and effective single copy detection of nucleic acid target sequences within a complex genome using optimised conditions and efficient primers. This work offers the potential for the development of digital LAMP for quantification without the requirement for standard curves to determine nucleic acid concentration.

## Methods

### Plant materials

Certified reference materials (CRM) for maize events Bt11 and NK603 from the Institute for Reference Materials (Geel, Belgium) purchased from Fluka GmbH (Buchs, Switzerland) were supplied with a defined mass fraction of 0.1 percent (Bt11) and 5.0 percent (NK603) w/w with wild type maize (0.1 percent Bt11: certified value 0.99 grams per kilogram with uncertainty of 0.13 grams per kilograms, 5 percent NK603: certified value 49.1 with uncertainty of 1.3). The maize event Bt11 developed by Syngenta Seeds S.A.S (Nerac, France) contains the transgenes *cry1Ab* (resistance to the European corn borer from *Bacillus thuringiensis* subspecies *kurstaki*) and *pat* (phosphinothricin N-acetyltransferase encoding gene from *Streptomyces viridochromogenes*, conferring tolerance to glufosinate herbicide). The expression of both transgenes is under the 35S promoter from the cauliflower mosaic virus (35Sp) and the nopaline synthase terminator from *Agrobacterium tumefaciens* (NOSt). Hence Bt11 contains two copies of both 35Sp and NOSt^46^. The maize event NK603, developed by Monsanto (St. Louis, United States) contains two copies of the transgene cp4epsps (5-enolpiruvilshikimate- 3-phosphate synthase from *Agrobacterium sp*. strain CP4 conferring glyphosate tolerance). The expression of one copy is promoted by 35Sp and both are terminated by NOSt. Seed stocks of commercial Mon810 were also used for DNA extraction after cleaning with molecular grade water to remove the anti-fungal coating and grinding to a fine powder with a mortar and pestle with liquid nitrogen. The maize event Mon810 produced by Monsanto contains the cry1Ab gene under the regulation of the 35S promoter and NOS terminator, although subsequent studies showed that the NOSt sequence has been lost from the Mon810 event^47,48^.

### Plant genomic DNA extraction

The Promega Wizard genomic DNA purification kit (Madison, United States) was used for the extraction of 40 mg of maize seed powder from the events Bt11, NK603 and Mon810 according to the manufacturer’s instructions for plant tissue. Extracts were rehydrated with 50 microlitres of the Tris:EDTA buffer from the kit and stored at 4 degrees C overnight.

### Plasmid DNA standard preparation

The pART7 plasmid^49^ containing the 35S promoter sequence was linearised by cutting with Eco RI. Initial quantification using the NanoDrop 1000 spectrophotometer and the Agilent 2100 Bioanalyzer preceded dilution with molecular grade water, salmon sperm DNA and gel loading buffer to give a final concentration of 4 × 10(6) copies per microlitre. Frozen 100 microlitre homogenised replicates were dried down in a centrifugal evaporator without heating and were re-suspended with 400 microlitre of molecular grade water to 10(6) copies per microlitre before use. Sequence data indicated a number of mismatches in this linearised plasmid to standard 35S promoter data and as a consequence the B3 displacement primer denoted K+ was redesigned and designated with (P).

### Purity and quantification of template DNA

The purity of the genomic DNA extracts from the maize events was analysed on 0.8 percent agarose gels prepared in TAE buffer, containing either SafeView (NBS Biologicals Ltd, UK) or GelRed (Biotium Inc., US) intercalating nucleic acid stains for visualisation using UV fluorescence in a Syngene U:Genius Gel Documentation system. The extracts were also assessed for purity using the NanoDrop 1000 spectrophotometer (Fisher Scientific, UK) using the wavelength ratios 260:280 nm and 260:230 nm. Values between 1.7 and 1.9 for the ratio 260:280 were deemed acceptable; as were those between 2.0 and 2.2 for the ratio 260:230.

Initial quantification of the template DNA was by NanoDrop® spectrophotometer to the manufacturer’s guidelines for double stranded DNA. Concordant quantification results were achieved between Qubit (Life Technologies, UK) fluorometric and agarose gel electrophoretic quantification. The Qubit dsDNA BR assay kit was used in accordance with the instructions with the Qubit 2.0 fluorometer. Agarose gel quantification used the method from Eurogentec using SmartLadder 10 kb and 0.8 percent agarose gels prepared in TAE buffer. The intensity of the gel bands of the ladder and samples were analysed using ImageJ (National Institutes of Health, USA) software version 1.51 u.

### Template denaturation

Where indicated, genomic and linear plasmid DNA templates were heat denatured at 95 degrees for five minutes before rapid cooling on ice for five minutes.

### Copy number calculations

**Figure 10:**
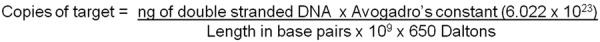
Copy Number Calculator.

The initial quantification values from NanoDrop, Qubit, agarose gel and Agilent Tapestation quantification methods were converted from nanogram per microlitre to copies per microlitre using the following formula in Fig. 10:

The maize genome was assumed to be 2.4 × 10(9) base pairs in length.

The online calculator was used at http://cels.uri.edu/gsc/cndna.html.

The CRM materials for maize events Bt11 and NK603, and the seed stock of Mon810 are hemizygous. Coupled with this the endosperm is triploid, one third of which is paternal and the embryo is diploid. Trifa and Zhang^36^ simulated the GM content for homozygous GM locus (385 copies per nanogram) and hemizygous with GM locus from male transgenic parent (160 copies per nanogram). In our calculations the transgenic copy numbers are adjusted by 160/385). Furthermore Bt11 has two copies of both the 35S promoter and the NOS terminator, doubling the available target sequences and the calculations of copy number reflect this. The calculations of copy number using the CRM material assume that the DNA content from both GM and non-GM samples combined will be the same. Environmental factors such as temperature and water content are also assumed to be the same.

### Primer design and synthesis

All oligonucleotide primers for LAMP and PCR DNA amplification were synthesized and supplied by Sigma (Poole, UK) to a standard level of purity (except where stated as HPLC purified). All primers were hydrated with molecular grade water to 100 micromolar and stored at −20 degrees. Primers used for qPCR and TaqMan digital PCR are displayed in Table 2 and LAMP primers are shown in Table 3.

**Table 2:** PCR Primers. Quantitative PCR primers which target the CaMV 35S promoter and NOS terminator sequences.

Target, Type, Notation, Version	Length	Tmelt	GC	Secondary	Primer Sequence (5′ to 3′)
35Sp, PCR, Forward, M3	21	63.1	42.9	Very Weak	CGTCTTCAAAGCAAGTGGATT
35Sp, PCR, Reverse, M3	22	65.6	45.5	Very Weak	TCTTGCGAAGGATAGTGGGATT
35Sp, TaqMan, Forward, M35 35SEF	23	67.2	43.5	Very Weak	CATCATTGCGATAAAGGAAAGGC
35Sp, TaqMan, Reverses, M35 35SER	21	67.8	47.7	Very Weak	TGCTTTGAAGACGTGGTTGGA
35Sp, TaqMan, Probe, M35 35SEP	15	64.7	73.4	None	(6FAM)TCGTGGGTGGGGGTC(OQA)
NOSt, PCR, Forward, HA-nos-118F	24	65.0	41.7	Very Weak	GCATGACGTTATTTATGAGATGGG
NOSt, PCR, Reverse, HA-nos-118F	24	73.9	54.2	Moderate	GACACCGCGCGCGATAATTTATCC

Version M3 denotes primers designed by Fernandez *et al*.^52^ and version HA-nos-118F and R denote primers designed by Lipp *et al*.^53^. TaqMan PCR primers targetting 35Sp were designed by Wu *et al*.^40^. HPLC grade purified primers were used unless specified. Data for Tmelt and secondary structure formation were derived from Sigma technical datasheets accompanying the primers.

**Table 3:** LAMP Primers.

Target, Type, Notation, Version	Length	Primer Sequence (5′ to 3′)
35Sp, Displacement, F3, DL	15	AGGAAGGGTCTTGCG
35Sp, Displacement, B3, DL	18	ATAAAGGAAAGGCCATCG
35Sp, LAMP, FIP, DL	39	GTCTTCAAAGCAAGTGG-TTTT-GGATAGTGGGATTGTGCG
35Sp, LAMP, BIP, DL	37	TTCCACGATGCTCCTCG-TTTT-CCTCTGCCGACAGTGG
35Sp, Loop, F-Loop, DL	16	TCCACTGACGTAAGGG
35Sp, Loop, B-Loop, DL	16	GGGGTCCATCTTTGGG
35Sp, Displacement, F3, CZ	18	AAGATGCCTCTGCCGACA
35Sp, Displacement, B3, CZ	19	CAGCGTGTCCTCTCCAAAT
35Sp, LAMP, FIP, CZ	39	ACGTGGTTGGAACGTCTTCTTCCCAAAGATGGACCCCCA
35Sp, LAMP, BIP, CZ	42	ATCTCCACTGACGTAAGGGATGATAGAGGAAGGGTCTTGCGA
35Sp, Loop, F-Loop, CZ	18	TCCACGATGCTCCTCGTG
35Sp, Loop, B-Loop, CZ	20	ACGCACAATCCCACTATCCT
NOSt, Displacement, F3, DL	22	CGCGATAATTTATCCTAGTTTG
NOSt, Displacement, B3, DL	19	CGTTCAAACATTTGGCAAT
NOSt, LAMP, FIP, PH	46	GCATGACGTTATTTATGAGA-TTTT-TCGCGCTATATTTTGTTTTCTA
NOSt, LAMP, BIP, PH	43	CATGCTTAACGTAATTCAACA-TTTT-TGAATCCTGTTGCCGGTC
NOSt, Loop, F-Loop, DL	22	GATTAGAGTCCCGCAATTATAC
NOSt, Loop, B-Loop, DL	23	AAATTATATGATAATCATCGCAA

Displacement, LAMP and loop primers which target the CaMV 35S promoter and NOS terminator sequences. Versions denoted with DL are primers designed by Lee *et al*.^28^ and version CZ refer to primers described in Zahradnik *et al*.^7^. NOSt FIP and BIP LAMP primers were modified to improve assay performance. HPLC grade purified primers were used unless specified.

### LAMP amplifications

The optimised chemistry for LAMP amplification was as described in Kiddle *et al*.^6^. Salmon sperm carrier DNA at 100 nanograms per partition was added to all LAMP reactions. The concentration of the primers for LAMP was 0.8 micromolar for each LAMP primer, 0.4 micromolar each Loop primer and 0.2 micromolar each displacement primer with a ratio of 4:2:1 respectively. Published concentrations of primers by Zahradnik *et al*.^7^ for the detection of the CaMV 35S promoter in GM crops, were 0.8 micromolar for each LAMP primer, 0.2 micromolar each Loop primer and 0.4 micromolar for each displacement primer with a ratio of 4:1:2 respectively. These differing concentrations and ratios were compared for LAMP assay target sensitivity. The LAMP assays were conducted on a programmable TRobot thermal cycler (Biometra, Göttingen, Germany) at a constant 60 degrees C for 100 minutes.

### Real-time and end-point detection

**Figure 11:**
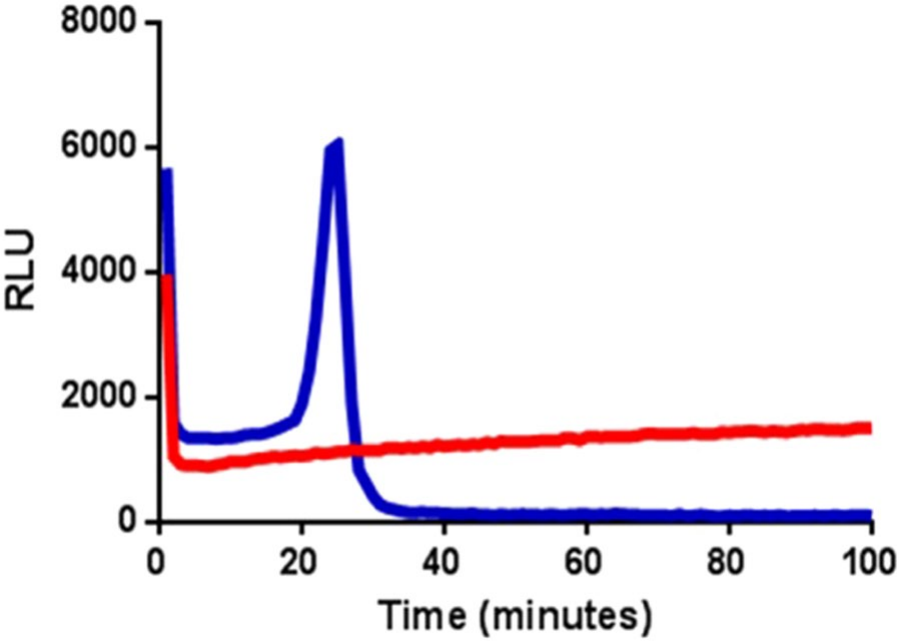
Bioluminescent Assay in Real Time. Bioluminescent light output of BART from nucleic acid isothermal amplification. Light output for a positive result from a partition containing DNA template in blue, negative result from a non-template control in red. The positive sample is characterised by a BART peak and the assay time at the maximum light intensity is defined as Tmax.

The LAMP-BART chemistry was previously described by Kiddle *et al*.^6^ and Gandelman *et al*.^26^. The final reaction volume for LAMP-BART assays was 20 microlitres inclusive of 5 microlitres of DNA template or negative template control. Assays were loaded to white 96 well microtitre plates, layered with mineral oil and sealed with a clear adhesive cover. The bioluminescent signal was recorded in a ‘LUCY’ developed by Lumora Ltd (Ely, UK), and analysed using React IVD software developed by Synoptics (Cambridge, UK) at 60 degrees C for 100 minutes. The BART detection system allowed the LAMP amplification of target DNA to be visualised in real time (Fig. 11).

End-point visualisation of LAMP was by resolution by electrophoresis on 2 percent agarose gels, prepared with TAE buffer, with GelRed intercalating nucleic acid stain and a UV transilluminator.

### Quantitative PCR amplifications

Samples were amplified by real-time quantitative polymerase chain reaction using a Qiagen (Hilden, Germany) Rotor-Gene thermal cycler and SYBR Green JumpStart Taq ReadyMix (Sigma; Poole, UK) according to the manufacturer’s recommendations. The reaction volume of 20 microlitres consisted of 5 microlitres of extracted DNA template or non-template control with molecular grade water, 10 microlitres of JumpStart, 5 picomoles of each respective primer (Sigma HPLC purified targeting the CaMV 35S promoter and the NOS terminator sequences) made up to volume with molecular grade water. The parameters were set at 94 degrees C for 2 minutes for activation of the hot-start Taq polymerase, followed by 50 cycles of 94C degrees (30 seconds), 50 degrees C (30 seconds) and 72 degrees C (30 seconds). Following amplification, the melting temperature of the amplicons was recorded between 60 and 92 degrees C. The Rotor-Gene 6000 software v1.7 and Microsoft Excel were used to analyse the data.

### Fluorescent LAMP amplifications

SYTO dyes have been successfully used in real-time PCR^50^ and real-time LAMP^51^ as an alternative to the more commonly used SYBR green dyes. Samples of a 4.9Kb linearised plasmid with the CaMV 35S promoter sequence were used to optimise a real time quantitative LAMP reaction (data not shown). The reaction volume of 20 microlitres contained 1x Isothermal Buffer (NEB), 300 micromolar each dNTP, 0.8 molar betaine, 0.5 micromolar SYTO 9 Green, 0.32 Units per microlitre Bst polymerase v2.0 WarmStart (NEB), 0.8 micromolar each LAMP primer, 0.4 micromolar each Loop primer and 0.2 micromolar each displacement primer, made up to the final volume with molecular grade water. The real-time fluorescent LAMP reaction was performed on the Corbett Rotor-Gene thermal cycler at 60 degrees C for 60 seconds for a total of 100 cycles acquiring fluorescence data during each cycle. The DNA amplification was followed by amplicon melt temperature recording between 60 and 92 degrees C. The Rotor-Gene 6000 software v1.7 and Microsoft Excel were used to analyse the data.

### Agilent Tapestation quantification

Maize genomic DNA from the event Bt11 were quantified on genomic tapes in the Agilent Tapestation (Agilent; California, USA) following the protocol for genomic tapes. Briefly 1 microlitre of either genomic DNA or ladder was combined with 10 microlitres of genomic DNA sample buffer, vortexed and loaded into the instrument. The genomic tapes were robotically loaded and the resulting bands sized between 200 and greater than 60000 base pairs.

### Digital PCR quantification

The 35S promoter sequence in maize event Bt11 was targeted with TaqMan PCR primers^40^ and a specific probe with 6-FAM fluorophore and OQA quencher at 5′ and 3′ ends respectively (Table 2). The QuantStudio 3D digital PCR system (Life Technologies; California, USA) was used according to the manufacturer’s instructions to load a reaction volume of 14.5 microlitres containing 7.25 microlitres QuantStudio 3D digital PCR mastermix, 0.4 micromolar each primer, 0.2 micromolar TaqMan probe, molecular grade water and template, to a chip for thermocycling. The fluorescent signal from each chip partition was acquired and the data was analysed with QuantStudio 3D Analysis Suite web software.

### “Accession codes”

EU760495.1. JX139718.1. KJ608135. KJ608143.1. KJ608144.1.

## Electronic supplementary material

Supplementary Information

## Data Availability

The datasets generated during and/or analysed during the current study are available from the corresponding author on reasonable request.
