# Supplementary material for: Optimised LAMP allows single copy detection of 35Sp and NOSt in transgenic maize using Bioluminescent Assay in Real Time (BART)
Source: Sci Rep. 2018 Dec 4;8:17590. doi: 10.1038/s41598-018-36207-4 (PMC6279926; doi:10.1038/s41598-018-36207-4)
Supplement: Supplementary file 1 — Supplementary Information [file 41598_2018_36207_MOESM1_ESM.docx]

Supplementary Information

**Optimised LAMP allows single copy detection of the 35Sp and NOSt in transgenic maize using the Bioluminescent Assay in Real Time (BART)**

**Patrick Hardinge^1,*^, Guy Kiddle^2^, Laurence Tisi^2^, James A. H. Murray^1^**

^1^Cardiff School of Biosciences, Biomedical Sciences Building, Museum Avenue, Cardiff CF10 3AX, UK

^2^ERBA MDX, Bartholomew Walk, Cambridgeshire Business Park, Ely, Cambridgeshire CB7 4EA, UK

[*hardingep@cardiff.ac.uk](mailto:*hardingep@cardiff.ac.uk)


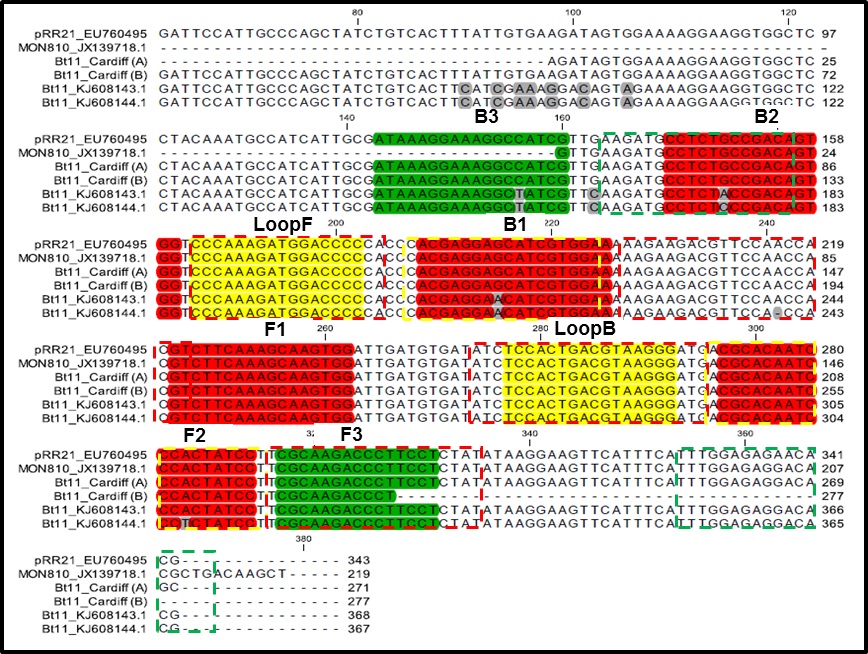


**Figure S1. Sequencing data for Bt11 35S promoter to show LAMP primer positions.**

New CaMV 35Sp sequence data from PCR amplified Bt11 transgene compared to GenBank EU760495.1, JX139718.1 and KJ608143/4 highlighting the partial nature of the 35S promoter sequence from JX139718.1. The alignments of the sequences are shown with the position of LAMP primers designed by Lee et al 2009 and used by Kiddle et al (hereafter “K primers”), highlighted as follows; displacement primers denoted F3 and B3 are in green, loop primers denoted by F-Loop and B-Loop are in yellow and the hairpin-forming LAMP primers FIP and BIP composed of the two sequences F2, F1c and B2, B1c as indicated and highlighted in red. Primers used by Zahradnik et al 2014 (“Z primers”) are also indicated with dashed lines.


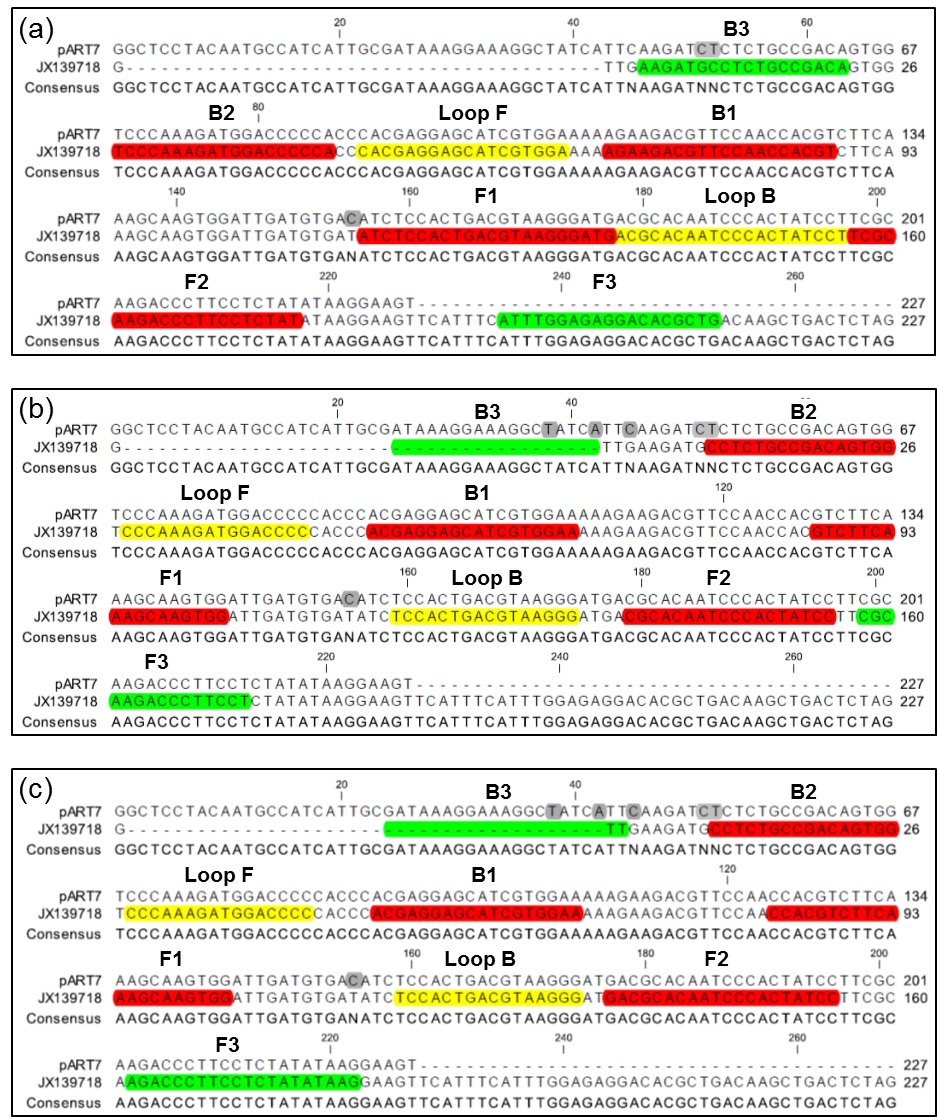


**Figure S2. Sequencing data for pART7 35S promoter to show LAMP primer positions.**

New CaMV 35Sp sequence data from plasmid pART7 compared to GenBank JX139718.1 and highlighting the position of LAMP primers; (a) Z-primers, (b) K-primers and (c) K+-primers, highlighted as follows; displacement primers denoted F3 and B3 are in green, loop primers denoted by F-Loop and B-Loop are in yellow and the hairpin-forming LAMP primers FIP and BIP composed of the two sequences F2, F1c and B2, B1c as highlighted in red. Non-consensus sequences highlighted in grey, K+-primer B3(P) redesigned for mismatches in pART7 sequence.


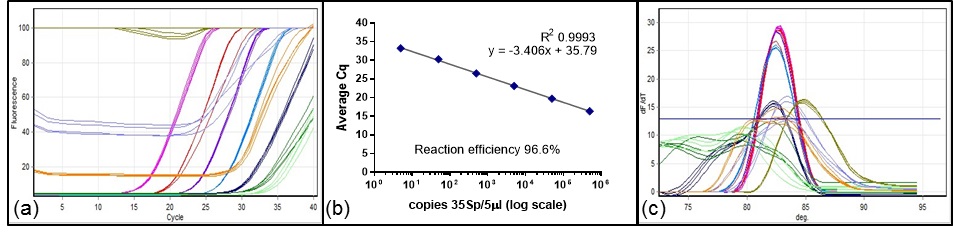


**Figure S3. Replicate of Quantitative PCR of 0.1 percent Bt11 DNA Extract.** Quantitation of Bt11 DNA extract using 35Sp primers, standard curve and qPCR. Standard curve using linear plasmid pART7 with defined copies of 35Sp sequence incrementally from 5 x 10(5) copies (pink) to 5 copies per 5 microlitres (dark green). The standard curve of the linear plasmid DNA has calculated PCR efficiency of 98.6 percent with R squared value of 0.9993. Dilutions of the 0.1 percent Bt11 extract were; mid-green for undiluted, mid-blue for 1:2 dilution and light brown for 1:5 dilution. NTCs shown in pale green.

**
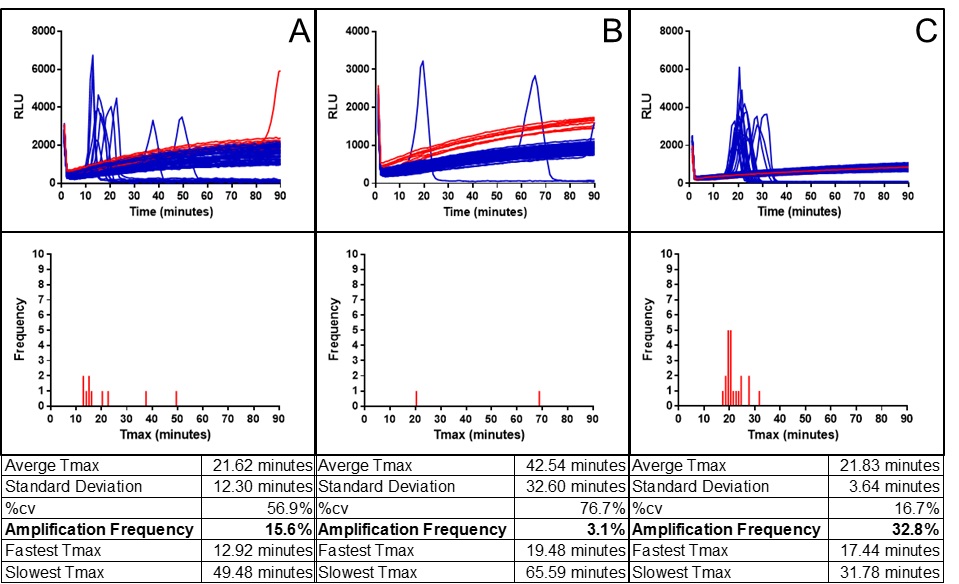
**

**Figure S4. Replicate LAMP-BART detection of 0.8 copies of 35S promoter or NOS terminator in denatured maize genomic Bt11 DNA extract.** Column A: K+-primer assay; column B: Z-primers; column C: NOSt primer assay. The top row shows traces from replicate partitions of LAMP-BART assays for 0.8 copies per partition (n=64) in blue and the NTCs (n=8) in red. The frequency distribution of LAMP-BART Tmax time-to-peak results for each assay is below with a summary analysis table.


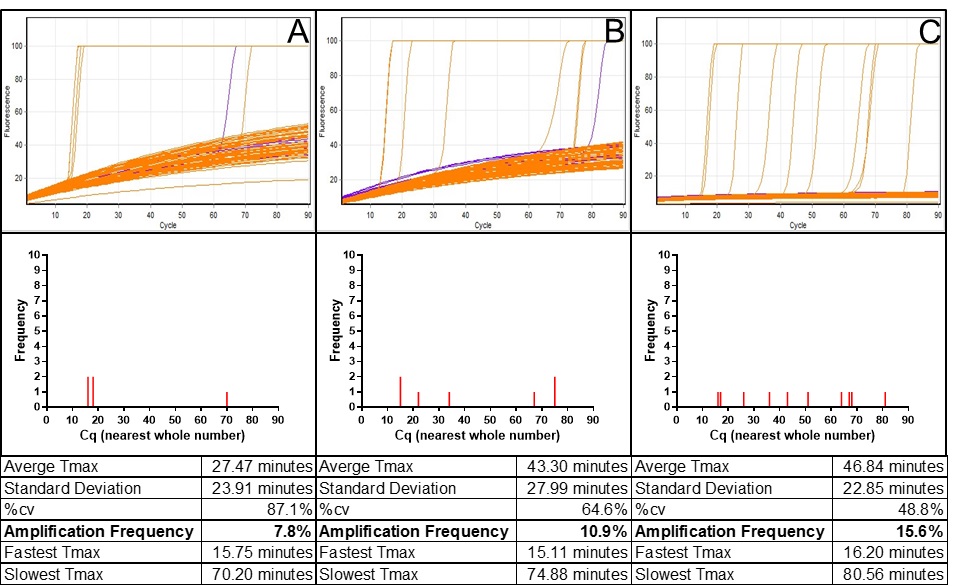


**Figure S5. Replicate Fluorescent LAMP detection of 0.8 copies of 35S promoter in denatured maize genomic Bt11 DNA extract.** Columns A and B: K+-primer assay; column C: Z-primer assay. The top row shows the fluorescent LAMP assays of 0.8 copies per partition (n=64) in orange and the NTCs (n=8) in red. The frequency distribution of fluorescent LAMP results for each assay is below with a summary table (each nominal cycle was 1 minute).


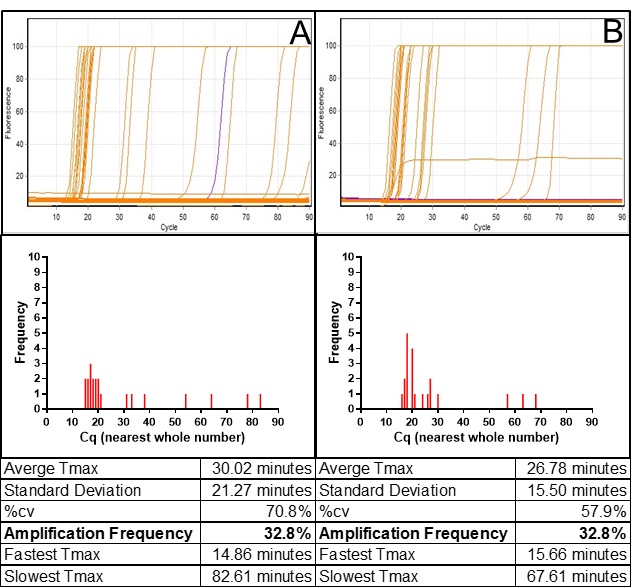


**Figure S6. Replicate Fluorescent LAMP detection of 0.8 copies of NOSt in Bt11 denatured DNA extract.** Columns A and B refer to NOSt primer assays. The top row shows the fluorescent LAMP assays of 0.8 copies per partition (n=64) in orange and the NTCs (n=8) in red. The frequency distribution of fluorescent LAMP results for each assay is below with a summary table (each nominal cycle was 1 minute).


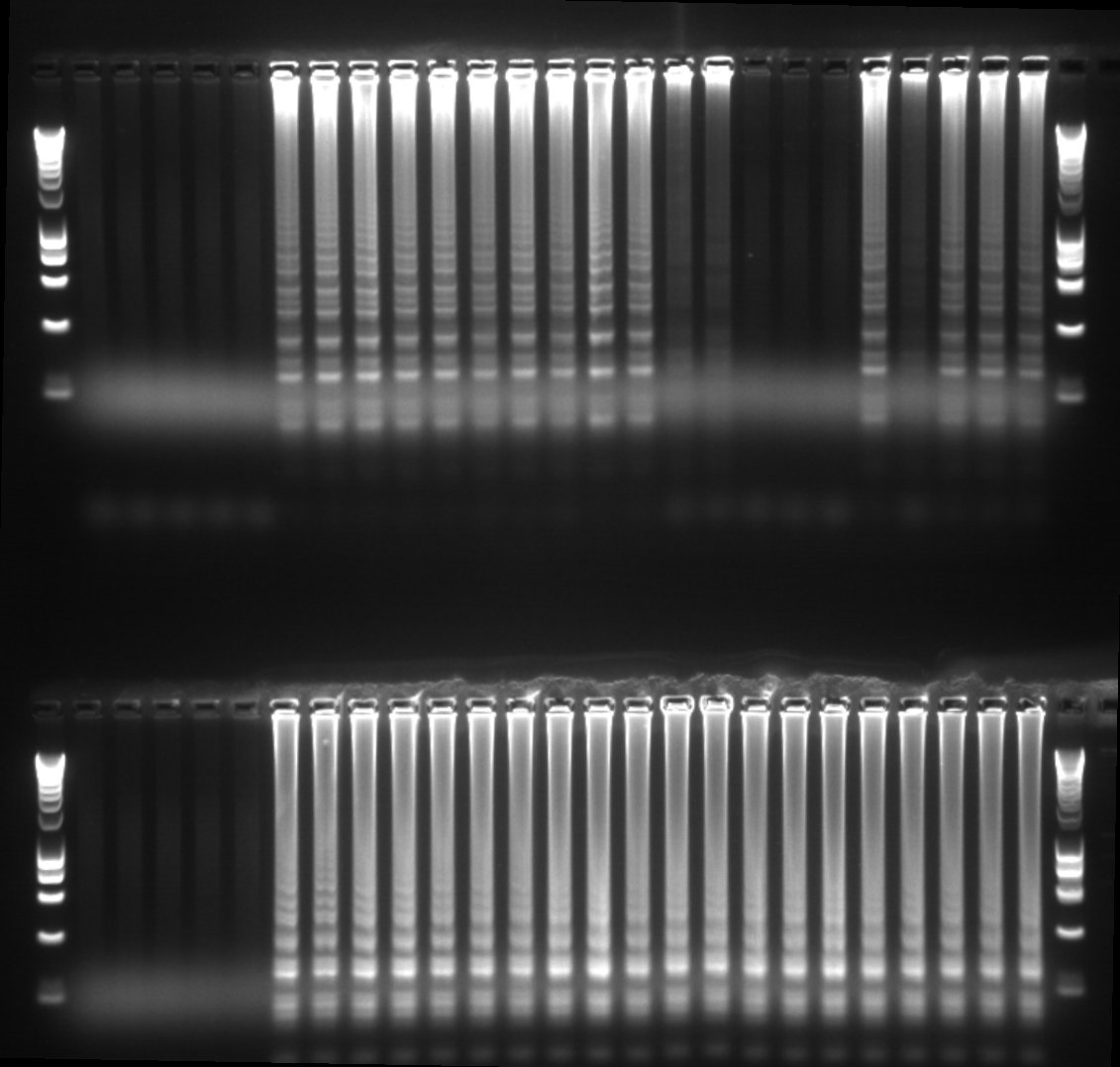


**Figure S7. Full length agarose gel used in Figure 4.** The upper section of the gel was used for panel (b) and the lower section for panel (f) in Figure 4.


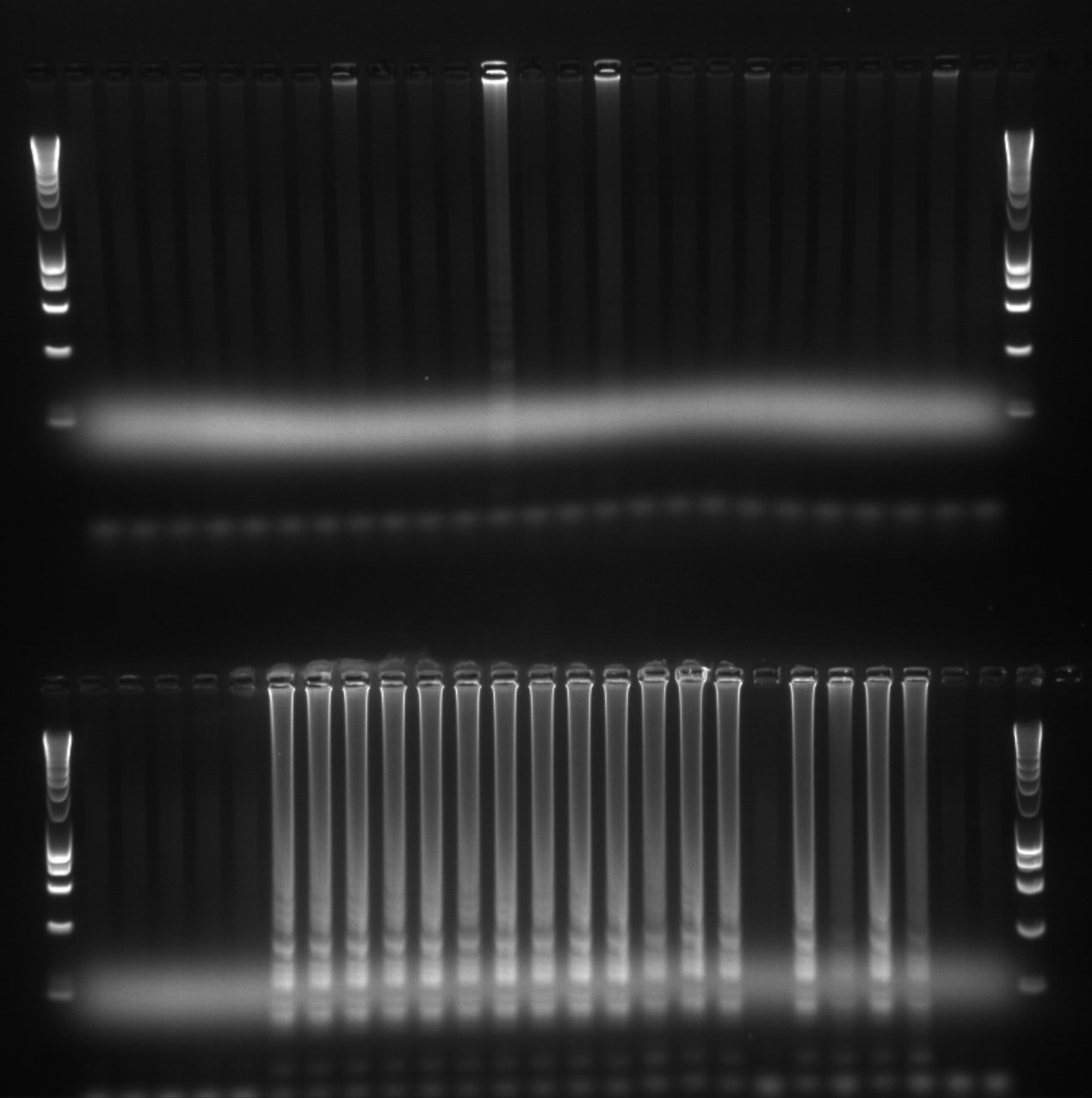


**Figure S8. Full length agarose gel used in Figure 4.** The upper section of the gel was used for panel (d) and the lower section for panel (h) in Figure 4.


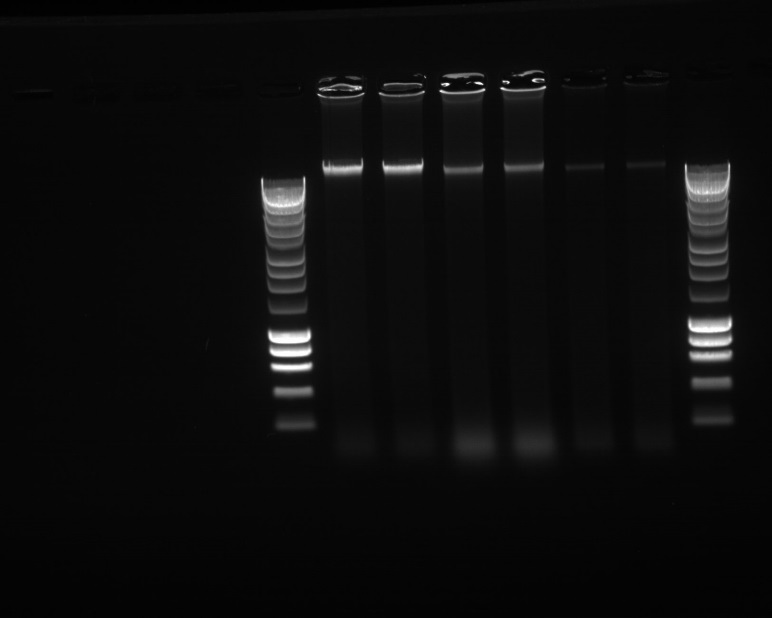


**Figure S9. Full length agarose gel used in Figure 5.**
